# A human urothelial microtissue model reveals shared colonization and survival strategies between uropathogens and commensals

**DOI:** 10.1126/sciadv.adi9834

**Published:** 2023-11-08

**Authors:** Carlos Flores, Jefferson Ling, Amanda Loh, Ramón G. Maset, Angeline Aw, Ian J. White, Raymond Fernando, Jennifer L. Rohn

**Affiliations:** ^1^ Centre for Urological Biology, Division of Medicine, University College London, WC1E 6BT London, UK.; ^2^ Laboratory for Molecular Cell Biology, University College London, WC1E 6BT London, UK.; ^3^ Royal Free London NHS Foundation Trust & Anthony Nolan Laboratories, NW3 2QG London, UK.

## Abstract

Urinary tract infection is among the most common infections worldwide, typically studied in animals and cell lines with limited uropathogenic strains. Here, we assessed diverse bacterial species in a human urothelial microtissue model exhibiting full stratification, differentiation, innate epithelial responses, and urine tolerance. Several uropathogens invaded intracellularly, but also commensal *Escherichia coli*, suggesting that invasion is a shared survival strategy, not solely a virulence hallmark. The *E. coli* adhesin FimH was required for intracellular bacterial community formation, but not for invasion. Other shared lifestyles included filamentation (Gram-negatives), chaining (Gram-positives), and hijacking of exfoliating cells, while biofilm-like aggregates were formed mainly with *Pseudomonas* and *Proteus*. Urothelial cells expelled invasive bacteria in Rab-/LC3-decorated structures, while highly cytotoxic/invasive uropathogens, but not commensals, disrupted host barrier function and strongly induced exfoliation and cytokine production. Overall, this work highlights diverse species-/strain-specific infection strategies and corresponding host responses in a human urothelial microenvironment, providing insights at the microtissue, cell, and molecular level.

## Teaser

Bacteria deploy varied interaction strategies in a human bladder microtissue model, eliciting specific host innate responses.

## INTRODUCTION

Urinary tract infection (UTI) is one of the most common human bacterial infections, with recurrence rates of ~30% within 6 months, highlighting the suboptimal performance of antibiotics (*1*). Because of successive treatment rounds, including prophylaxis, UTIs exacerbate the global antimicrobial resistance crisis (*2*, *3*). Together with notable morbidity, reduced life quality, and mortality, UTI represents a hefty economic burden for health care systems worldwide (*1*).

Mouse models and murine/human bladder or kidney cell lines have enabled advances in understanding host-uropathogen interactions, especially in the case of uropathogenic *Escherichia coli* (UPEC), one of the most common and well-studied culprits (*4*). After attachment to mouse bladders or urothelial cells (e.g., T24, 5637, SV-HUC-1), a number of studies established a key virulence role for bacterial invasion and intracellular bacteria communities (IBCs), which, in turn, can assume a pod-like aspect that erupts, releasing filamentous bacterial forms; in parallel, exfoliation of the upper cell layers allows deeper bacterial access and establishment of quiescent intracellular bacterial reservoirs (*5*). However, outside of these model systems, to our knowledge, only one study has reported intratissue/intracellular bacteria directly in the bladder wall of patients (*6*), with others having identified them sporadically in shed urothelial cells from patient urine (*7*–*10*). Hence, a key question in the field is how common intracellular invasion is in humans and how it is achieved. Several virulence factors have been identified so far, especially uropathogenic fimbriae, such as the widely studied type 1 pilus and its tip adhesin, FimH, which has been implicated in multiple infection steps (*5*, *11*–*13*). Therefore, FimH has served as a popular target for the development of antibiotic alternatives over the past decades, but, again, further studies in advanced human cell settings would be illustrative (*14*, *15*).

Matters are even less certain for other UPEC besides the commonly studied UTI89 and CFT073 strains, or with nonpathogenic urocolonizing bacteria, either commensals isolated from the bladder or urine of uninfected individuals, or asymptomatic bacteriuria (ASB) strains such as *E. coli* 83972 (HM50), which grow to high levels in the bladder without causing signs or symptoms of infection. Some studies in vitro, in two-dimensional (2D) systems, or the mouse model, mainly using HM50, have shown not only genotypic and phenotypic differences between UTI and non-UTI strains but also great heterogeneity, including between commensals and ASB isolates (*16*–*20*). Moreover, other species besides *E. coli* are not rare, especially in older people, where polymicrobial infections abound, as well as in hospitals and catheterized patients, where *Pseudomonas aeruginosa* (PA), *Proteus mirabilis* (PM)*, Enterococcus* sp., and *Klebsiella pneumoniae* (KP) thrive.

Given that infected shed patient cells cannot reveal what is happening in deeper urothelial layers and the ethical issues involved in taking urothelial biopsies from infected patients, advanced human cell models are needed to help fill these gaps in our understanding of UTI. Urothelial stratification, differentiation, and the presence of apical urine create a unique interface for invading pathogens, which cannot be emulated using human cancer cell lines. Meanwhile, although the main animal model used in the field, the mouse, presents obvious benefits, key ultrastructural and physiological species differences exist between murine and human urothelium, including thickness and number of cell layers, tissue differentiation biomarkers, innate immunity players, and urine concentration (*21*). These issues have triggered the recent development of several human cell–based 3D urothelial models and organoids as a complementary approach (*22*–*25*), although most cannot mimic all of these human urothelial features.

Here, we deployed a robust 3D microtissue model [3D urine-tolerant human urothelial (3D-UHU)], recapitulating the critical human urothelial features including full human stratification and differentiation, alongside an innate epithelial immune response, enabling experiments in a 100% human urine environment to study the broader aspects of uropathogen-host interactions in multiple species. We asked (i) whether distinct UPEC clinical isolates and FimH adhesin mutants could invade this model and form IBCs; (ii) how commensal *E. coli* colonizes this environment; (iii) which strategies are used by Gram-positive versus Gram-negative uropathogens; and (iv) how the human microtissue responds to both uropathogens and commensals. Our results revealed an array of time-, species- and strain-specific diversity, with shared strategies between pathogens and nonpathogens (e.g., invasion and hijacking of exfoliating cells). Moreover, the host response usually correlated with specific bacterial strategies such as invasion or tissue barrier disruption and exfoliation. Overall, this model provides insights into human host-uropathogen interactions in a complex microenvironment, at both cellular and urothelial tissue levels.

## RESULTS

### UPEC displays strain-specific morphology and invasion phenotypes in a human urothelial microenvironment

**Fig. 1.**
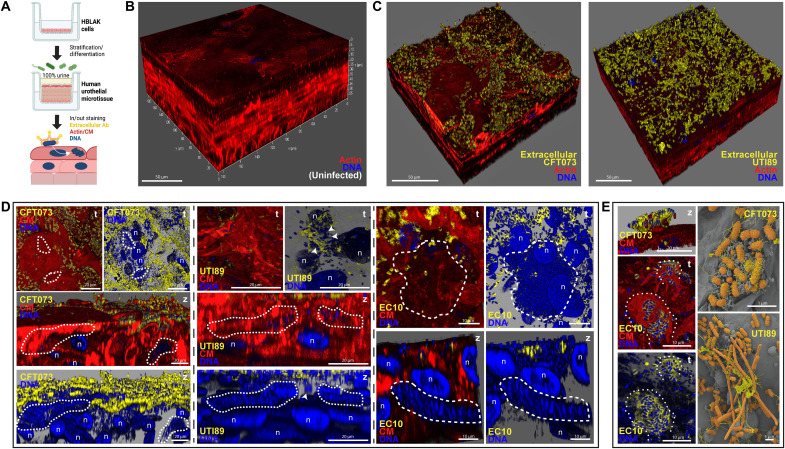
UPEC infection in the human urothelial model 3D-UHU. (**A**) Schematic representation of 3D-UHU development and staining for extracellular versus intracellular bacteria. Ab, antibody. (**B**) 3D view of uninfected model. (**C**) 3D views after 12 hpi with UPEC CFT073 and UTI89 (in yellow). (**D**) Invasion and IBC formation (dashed lines) by UPEC CFT073 (left), UTI89 (middle), and EC10 (right), 12 hpi at an MOI of 50. Arrowheads depict intracellular unaggregated UTI89. (**E**) UPEC IBC eruption at 12 hpi and MOI of 50. Dotted lines depict pod-like structures exposing EC10, and sprouting filaments can be observed for UTI89. t, top-down view; z, side view. Yellow, extracellular bacteria; blue, DNA of host nuclei (n) and bacteria (intracellular and extracellular); red, F-actin or cell membrane (CM). Confocal [(B) to (D) and (E), left] and scanning electron microscopy (SEM) [(E), right] images representative of a minimum of four independent biological replicates per strain (*N* ≥ 4).

**Fig. 2.**
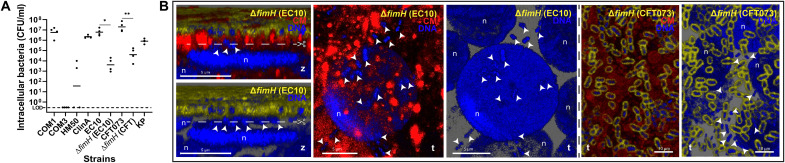
Bacterial invasion and UPEC ∆*fimH* phenotypes. (**A**) Intracellular bacteria quantified by gentamicin protection assay 12 hpi with nonpathogenic *E. coli* (COM1, COM3, and HM50), UPEC (ClinA, EC10, CFT073, and respective ∆*fimH* mutants), and KP at an MOI of 100 [limit of detection (LOD) = 0 CFU/ml]. Data plotted as mean of four independent biological replicates (*N* = 4), **P < 0.01; *P < 0.1. (**B**) Invasion without IBC formation by ∆*fimH* in EC10 and CFT073 backgrounds (arrowheads). Scissors depict the position of cross sections for the side views for ∆*fimH* EC10 in the adjacent panels. Staining as in Fig. 1. Confocal images representative of a minimum of four independent biological replicates per strain (*N* ≥ 4).

We generated 3D-UHU microtissue models (Fig. 1, A and B), recapitulating key human features (fig. S1, A to D) (*25*): (i) thickness (~60 μm) in stratification, i.e., seven to eight cell layers; (ii) differentiation of the three main urothelium sublayers—basal, intermediate, and apical umbrella cells (expressing terminal differentiation markers); (iii) apical production of glycosaminoglycans (GAG layer); (iv) robust barrier function; and (v) 100% urine tolerance for long time periods (tested up to 1 month). We first examined the behavior of UPEC during infection in urine, testing well-known cystitis (UTI89) and pyelonephritis (CFT073) strains (Fig. 1C), alongside distinct clinical UTI isolates (table S1), namely, EC10 and EC6 (pandemic clones ST131) and ClinA. As expected, although fitness was somewhat impaired in urine only, all strains could grow and maintain their population (fig. S2A). In 3D-UHU, bacteria were mainly scattered on the apical urothelial surface after 3 hours after infection (hpi), while the population also increased over time (fig. S2B). From 6 hpi, CFT073, UTI89, and EC10 began to invade, accompanied by the formation of large communities (IBCs) that could later erupt, mainly after 12 hpi at a multiplicity of infection (MOI) of 50 to 100 (Fig. 1, D and E), with intracellular bacteria reaching >10^6^ colony-forming units (CFU)/ml as assessed by antibiotic protection assay (Fig. 2A). In contrast, ClinA and EC6 were able to invade but remained largely unaggregated, as intracellular isolated bacteria (fig. S2C) with lower bacterial loads (≤10^6^ CFU/ml; Fig. 2A). Sporadically, some biofilm-like aggregates were found on the 3D-UHU apical surface, mainly with UTI89 (fig. S2D), while elongated bacteria and filaments were found for all UPEC tested, sometimes accompanied by the eruption of IBCs (Fig. 1E).

**Fig. 3.**
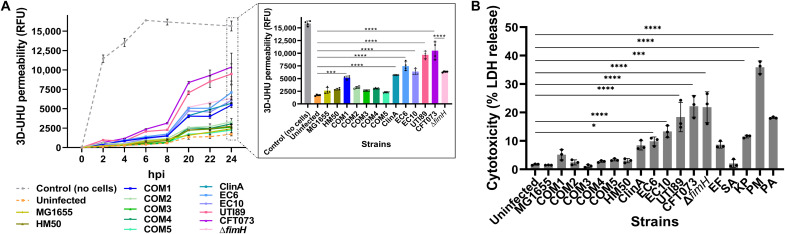
Bacterial effect on 3D-UHU tissue integrity. (**A**) 3D-UHU barrier function assessed by fluorescein isothiocyanate (FITC)–dextran (4 kDa) permeability assay before/after infection. Relative fluorescence units (RFU) were measured in basal chambers over 24 hours; data plotted as means ± SE of biological quadruplicates (*N* = 4). Inset compares the final time point. ****P < 0.0001; ***P < 0.001. (**B**) Cytotoxicity caused by the uropathogens and non-UPEC used in this study, 12 hpi, as assessed by lactate dehydrogenase (LDH) release assay. Data plotted as means ± SE of biological triplicates (*N* = 3). ****P < 0.0001; ***P < 0.001; *P < 0.1.

Urothelial damage caused by UPEC matched their invasive behaviors, with CFT073, closely followed by UTI89, having the strongest impact: a ~6-fold increase in barrier permeability (Fig. 3A) and ~20% more cytotoxicity compared to uninfected controls (Fig. 3B). In contrast, clinical UPEC isolates EC6 and ClinA had a weaker impact on both cytotoxicity (~8%) and permeability (three- to fourfold change).

### UPEC FimH is important for IBC formation but not essential for invasion

As the UPEC FimH adhesin was shown to be critical for the different stages of infection in cell lines and mice (*5*, *12*), we investigated its role for two distinct O-antigen UPEC serotypes, CFT073 (O6) and EC10 (O25b) in our model. Knockouts of the FimH adhesin showed invasion (~10^4^ CFU/ml), although this was significantly impaired compared to the parental strains (Fig. 2A), but neither mutant formed large IBCs, instead remaining largely isolated when intracellular (Fig. 2B). Moreover, filamentation was not affected in the mutants, but CFT073 ∆*fimH* caused less impairment of 3D-UHU permeability (~3- to 4-fold increase; Fig. 3A), despite causing similar cytotoxicity compared with the parental strain (Fig. 3B).

### Commensal *E. coli* shares survival strategies with UPEC, including invasion, filamentation, and hijacking of exfoliating cells

**Fig. 4.**
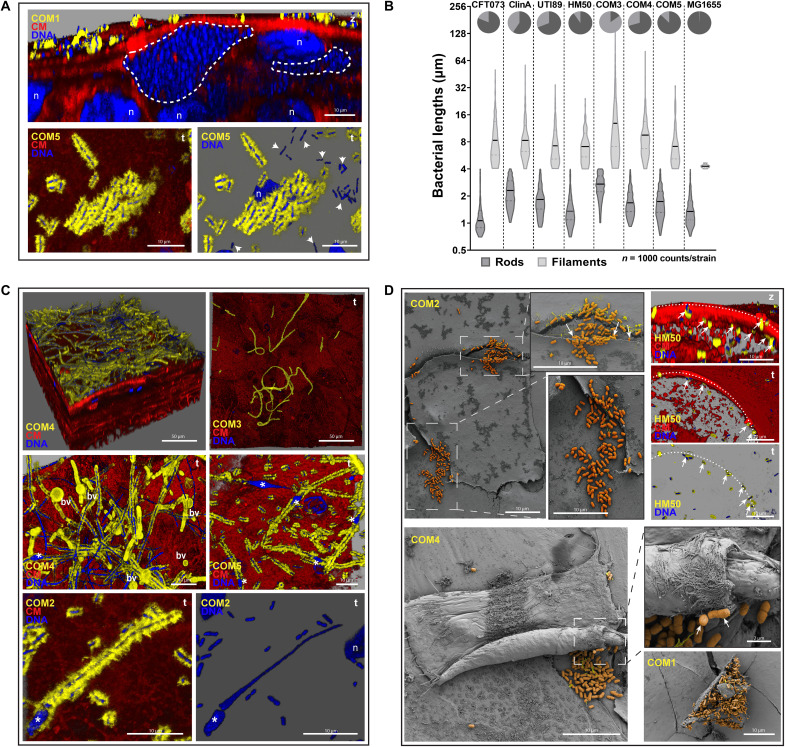
Non-UPEC colonization strategies and morphology in 3D-UHU. (**A**) Invasion by commensals at 12 hpi, COM1, with IBC formation (dashed lines), and COM5, isolated intracellularly (arrowheads). Staining as in Fig. 1. (**B**) Size distribution of *E. coli* at 12 hpi. Black lines, means of rod (dark gray) and filament (light gray) sizes; pie charts, proportion of each group in *N* = 1000 bacteria per strain. (**C**) Filamentation by commensal *E. coli* COM2, COM3, COM4, and COM5 accompanied by bacterial membrane vesiculation (bv) and fusiform nuclei (*) at 12 hpi. (**D**) Adhesion by COM2, HM50, and COM4 to the underside of exfoliating cells. Dotted lines depict the edge of cell with HM50 underneath (arrows). Bottom right: COM1 surrounding a dying cell. Confocal [(A), (C), and (D), top right] and SEM [(D), left and bottom] images representative of a minimum of four independent biological replicates per strain (*N* ≥ 4).

Intracellular invasion of UPEC has long been considered a hallmark of virulence in mouse models and cell lines (*5*). We interrogated this in our human microtissue model using the laboratory strain MG1655, the well-characterized ASB strain HM50, and five genetically distinct commensal *E. coli* isolated from clean-catch midstream urine samples of uninfected volunteers, hereafter referred to as “commensals” for convenience: COM1 to COM5 (table S1). COM1 and COM5 could invade umbrella cells, albeit differently: COM1 invaded frequently (~10^7^ CFU/ml; Fig. 2A) with IBC formation comparable to some UPEC (Fig. 4A, top), while COM5 invaded only sporadically and remained isolated without forming large communities (Fig. 4A, bottom, and fig. S2C). We also detected very rare invasion events by HM50, mostly in exfoliating cells, ranging from 0 to ~10^3^ CFU/ml (Fig. 2A). This finding also supports FimH not being essential for invasion, as HM50 does not produce a functional type 1 pili (*17*). Most of the other commensal bacteria were also sparsely piliated (fig. S3). COM2, COM3, COM4, and MG1655 were only observed on the apical surface, with no intracellular COM3 detected by gentamicin protection assay (Fig. 2A).

On the other hand, filamentation was extremely common among the non-UPEC strains, observed from 3 to 24 hpi, even in noninvasive strains (Fig. 4, B and C). Mean bacterial lengths (~1- to 2-μm rods and ~8- to 10-μm filaments) were similar among UPEC and non-UPEC, but with strain-specific variation in the proportion of filaments versus rods (Fig. 4B). For COM3, the bacterial population was almost entirely filamentous (~85%), with longer mean sizes (>3-μm rods and >9-μm filaments), but they poorly adhered and were easily washed away. Despite encoding the FimH adhesin, COM3 probably lacks functional type 1 pili and other fimbriae due to mutations detected after sequencing *fim* and other operons, similarly to HM50, and as supported by their lack of hemagglutination ability (fig. S4). Moreover, filamentation by non-UPEC on top of the undamaged umbrella cell layer was often accompanied by bacterial nucleoid enlargement, e.g., into a fusiform shape, and membrane vesiculation (Fig. 4C). Unlike UPEC, most non-UPEC caused negligible damage to 3D-UHU, with the exception of the IBC-forming COM1, which induced moderate loss of barrier permeability (Fig. 3A), but no significant cytotoxicity (Fig. 3B). However, a common phenotype among *E. coli* was adherence/colonization of the underside of cells being exfoliated and/or dying (Fig. 4D).

### Gram-positive and Gram-negative uropathogens exhibit specific behaviors in a human urothelial microenvironment

**Fig. 5.**
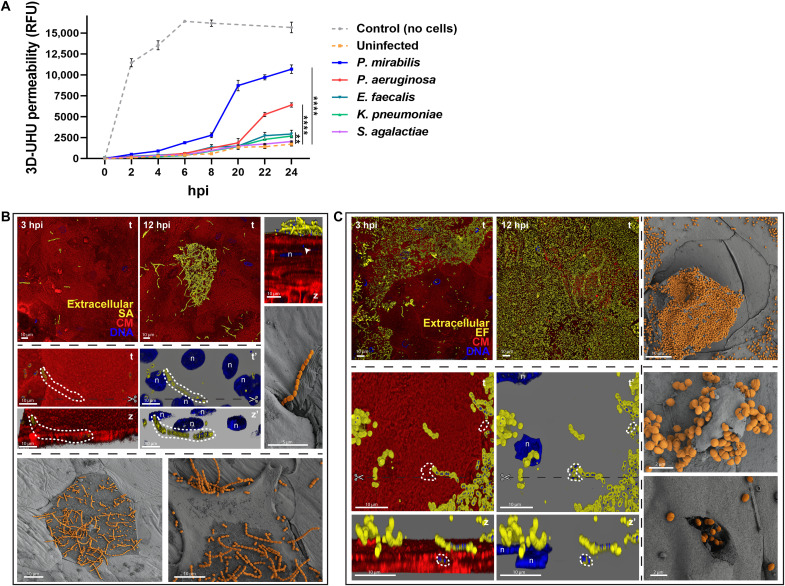
Effect of non-UPEC uropathogens on 3D-UHU permeability and infection by Gram-positive uropathogens. (**A**) 3D-UHU barrier function after infection with non-UPEC uropathogens, assessed by FITC-dextran (4 kDa) permeability assay. Fluorescence measured in basal chambers over 24 hours. Data plotted as means ± SE of biological triplicates (*N* = 3; ****P < 0.0001; **P < 0.01). (**B**) SA in discrete regions of the urothelial surface and associated with damaged upper cell host membranes (bottom). Cocci invasion (arrowhead at right uppermost panel) and chains underneath upper cell layers (middle, dotted lines). Scissors depict the place of cross sections for the side views in the adjacent bottom panels. (’) represents images without the red channel. (**C**) EF spread on the urothelial surface and chains translocating between the upper cell layers (bottom, dotted lines). Heavily colonized umbrella cells being exfoliated (right uppermost) and cocci erupting from a vesicle-like structure and urothelial cell (right bottom). Staining as Fig. 1. Confocal [(B), top and middle, and (C), left] and SEM [(B), middle right and bottom, and (C), right] images representative of a minimum of four independent biological replicates per strain (*N* ≥ 4).

**Fig. 6.**
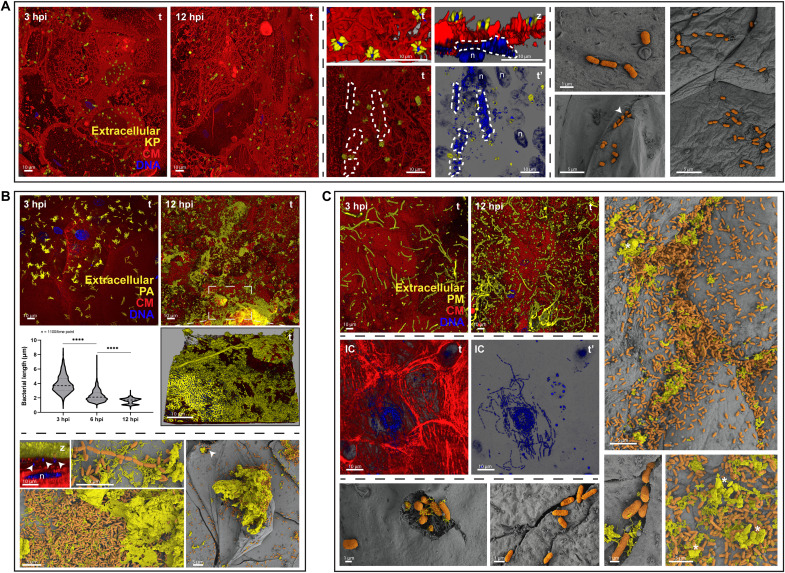
Invasion and biofilm formation by Gram-negative non-UPEC uropathogens in the 3D-UHU. (**A**) KP infection. Bacteria scattered on the urothelial surface (at 3 and 12 hpi); membrane ruffling and spike-like structures surrounding KP. Dashed lines depict IBCs (middle); arrowhead depicts possible eruption (bottom right). Staining as in Fig. 1. (’) represents images without the red channel. (**B**) PA infection. Formation of biofilm-like aggregates, incorporating cell debris, and precipitates (bottom SEM images). Violin plot showing decrease in bacteria length over course of infection (*N* = 1100 bacteria per time point; ****P < 0.0001). Arrowheads in bottom panels depict intracellular bacteria. (**C**) PM infection. Rods, chains, and/or elongated forms on 3D-UHU surface (top), inside a cross section of intermediate cell layers (IC), inside umbrella cells (bottom left), and penetrating paracellularly in inflamed urothelium while forming interjunctional biofilms with crystalline precipitates (*) (right and bottom SEM images). Confocal [(A), left and middle; (B), top; and (C), left top and middle] and SEM [(A), right; (B), bottom; and (C), right and bottom] images representative of a minimum of four independent biological replicates per strain (*N* ≥ 4).

Given the diversity seen within *E. coli*, we next inspected the behavior of other common uropathogens in the 3D-UHU microenvironment: the Gram-positive *Enterococcus faecalis* (EF) and *Streptococcus agalactiae* (SA), and the Gram-negatives KP, PA, and PM. First, PM impaired urothelial barrier function even more markedly than UPEC did by 84% (Fig. 5A) alongside a ~35% increase in cytotoxicity (Fig. 3B). PA and KP caused less cytotoxicity (18 and 14%, respectively), comparable to most UPEC, followed by EF, while SA showed the weakest impact on 3D-UHU, comparable to the uninfected control (Figs. 3B and 5A). In addition, all pathogens colonized vast areas of the urothelial surface, except SA (Fig. 5B) and KP (Fig. 6A), which were sparsely distributed even after long periods. KP was found in host membrane ruffles or surrounded by spike-like protrusions (Fig. 6A). With SA, discrete chain niches were found 6 hpi, as small aggregates on top of umbrella cells with damaged plasma membranes (Fig. 5B and fig. S5A).

The formation of chains was a common strategy among uropathogens, although with time- and species-dependent heterogeneity. Gram-positives formed longer chains from 3 to 12 hpi with increasing amounts of isolated cocci over time (Fig. 5, B and C), while PA (Fig. 6B) and PM chains (Fig. 6C) tended to be longer 12 hpi. In addition, both Gram-positive (Fig. 5, B and C) and PM (Fig. 6C) chains were able to translocate paracellularly, mainly between cells undergoing exfoliation, and, in case of PM, to access deeper layers and reside in between/inside intermediate cells (Fig. 6C, middle). For PA, long-chain formation coincided with the development of larger biofilm-like aggregates that merged and thickened over time, often containing cell debris and covering wide portions of the urothelial surface by 12 hpi (Fig. 6B and fig. S5B). This was accompanied by the decrease in PA size, from ~4 down to ~1 μm (Fig. 6B). In addition, PA and PM biofilms usually contained precipitates with a crystalline aspect, and, in the case of PM, these deposits were particularly larger, more abundant and interjunctionally positioned (Fig. 6C, right, and fig. S5C). Moreover, PA and PM produced more biofilms in urine or optimal medium alone compared with the other uropathogens, approximately 6× higher for PA, despite the general negative impact of urine on biofilm production (fig. S5D).

Intracellular invasion was commonly observed for KP and PM, as for some UPEC after 6 hpi, including in intermediate cells for PM after paracellular penetration and with formation of large IBCs in umbrella cells by KP (Fig. 6A, middle). All the remaining uropathogens could also invade, but this was rare and only seen after 12 hpi (e.g., Figs. 5, B and C, and 6B). Similar to *E. coli*, adhesion to the underside of cells undergoing exfoliation/dying was common among all non-UPEC uropathogens (Figs. 5C and 6B and fig. S5, C and E).

### A battery of tissue innate and autonomous urothelial responses differentially target uropathogens versus nonuropathogens

**Fig. 7.**
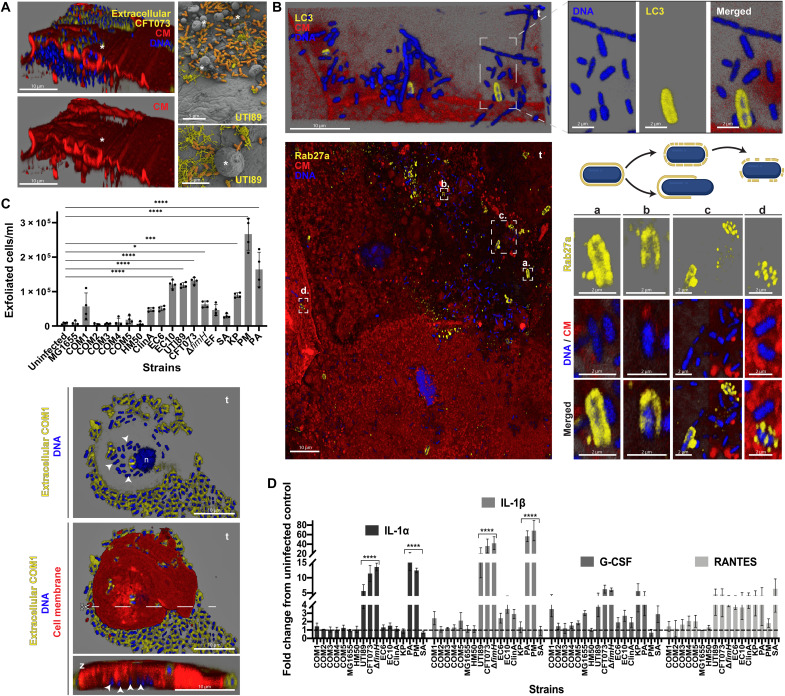
Host responses to uropathogens and commensals. (**A**) Membrane ruffling and formation of blebs/vesicle-like structures (*) by umbrella cells 12 hpi with CFT073 (left) and UTI89 (right). Staining as in Fig. 1. (**B**) Expelled CFT073 in exosome-like structures decorated with LC3 (top) and Rab27a (bottom). a to d depict different encasement patterns suggesting bacterial release either through elongation or case degradation (schematics above the images). (**C**) Host cell exfoliation 12 hpi with uropathogens and nonuropathogenic bacteria, and exfoliated cell with intracellular COM1 (arrowheads on images below). Staining as in Fig. 1. Scissors depict cross-sectional placement for the side view, shown at bottom. Data plotted as means ± SE of biological quadruplicates (*N* = 4); ****P < 0.0001; ***P < 0.001; *P < 0.1. (**D**) Cytokine (IL-1α, IL-1β, G-CSF, and RANTES) production by 3D-UHU 12 hpi. Fold changes compared to uninfected controls (dashed line) plotted as means ± SE of biological triplicates (*N* = 3); ****P < 0.0001. Confocal [(A), left, (B), and (C)] and SEM [(A), right] images representative of a minimum of four independent biological replicates per strain (*N* ≥ 4).

We frequently observed the extrusion of host vesicle-like structures and/or cell fragments of varying sizes from umbrella cells during infections (Fig. 7A and fig. S6A). With SA, these structures were usually devoid of bacteria but heavily surrounded by long chains (fig. S6A). In contrast, infections with more invasive strains (e.g., CFT073, UTI89, and COM1) featured bacteria inside the vesicle-like structures (fig. S6, A and B), often localized in areas with extensive plasma membrane ruffling (Fig. 7A and fig. S6C).

We also detected encasement of UPEC in “exosome-like” structures containing the phagosome marker LC3 (Fig. 7B). In addition, Rab27a (related to exocytosis and vesicle trafficking) also colocalized with extracellular UPEC, commensal *E. coli*, and KP (e.g., for CFT073 in Fig. 7B). These structures were not homogeneous, varying from a complete “cage” to porous structures or protein patches. Rab27b (a marker of fusiform vesicles) was also observed in these structures, but as it was highly expressed throughout the umbrella cell layer, specificity for encased bacteria was not so obvious and therefore was not assessed.

Sporadic umbrella cell exfoliation, a homeostatic urothelial mechanism (*26*), could be reproduced by uninfected 3D-UHU (Fig. 7C). However, more exfoliation occurred during infection with aggressive strains, such as with the invasive PM (~3 × 10^5^ exfoliated cells/ml), CFT073, UTI89, and EC10 (~1 × 10^5^ exfoliated cells/ml), as well as the biofilm former PA (~1.5 × 10^5^ exfoliated cells/ml). With IBC formers, these exfoliated cells enclosed frequently bacteria inside (Fig. 7C). In contrast, significant exfoliation did not occur for non-UPEC strains, even with the efficient invader COM1, compared to uninfected controls.

The apparent specificity in host responses against uropathogens was also extended to cyto/chemokine production (Fig. 7D). Among the 16 analytes assessed, interleukin-1β (IL-1β) production was by far the most accentuated, especially with UTI89, CFT073, PA, and PM (>10-fold higher), followed by IL-1α, granulocyte colony-stimulating factor (G-CSF), and RANTES. Increase in IL-6, IL-8, and Pentraxin 3 against uropathogens was also detected (fig. S6D). The proinflammatory reaction agrees with the distended aspect of upper cell layers suggestive of urothelial inflammation, e.g., in PM (Fig. 6C and fig. S5C, right). Unexpectedly, infections with PM or PA also induced similar or much lower levels of some cytokines compared with nonpathogenic strains or uninfected controls, with IL-6 production being particularly affected (fig. S6D). Moreover, in agreement with the abovementioned data, CFT073 and UTI89 triggered the most pronounced response among UPEC, while most nonpathogenic *E. coli* induced a response similar to uninfected controls, the weakest being HM50 and COM3 (Fig. 7D and fig. S6D). No significant differences among all the bacteria studied were observed for Lactoferrin and Lipocalin-2, whereas osteopontin and interferon-γ were not detected.

## DISCUSSION

Two decades after the first ground-breaking report of intracellular UPEC in the murine UTI model (*27*), many questions remain about host-pathogen interactions in the human bladder. Driven by the need for advanced human-derived models (*21*), we developed 3D-UHU as a robust platform mimicking critical human urothelial features (*25*). The long-term tolerance of urine is key, as it affects not only host cells (*28*) but also bacterial growth, metabolism (*29*), pathogenicity, e.g., biofilm formation (fig. S5D) (*30*), and virulence factor expression (*31*). Moreover, the multiple intermediate cell layers, as opposed to just one in mice (*32*), are critical for assessing the establishment of deeper bacterial reservoirs and regeneration after exfoliation, as well as the balance between recurrence and UTI resolution in humans.

Using this human-relevant microenvironment, with undiluted urine, we investigated various uropathogens alongside commensal or ASB *E. coli* and observed a wider diversity of time-, species- and (as shown for *E. coli*) strain-specific behaviors compared with what is documented for particular animal or in vitro models, which often featured a more restricted set of strains, mainly UPEC. In mice, UPEC cause stereotypical IBCs (*27*), which could be recapitulated in 3D-UHU with some clinical isolates. However, two other clinical strains invaded without forming IBCs, remaining as isolated bacteria. In addition, the commensal strain COM1, isolated from the urine of a healthy volunteer, invaded frequently and formed large IBCs, while other nonpathogenic strains also invaded, albeit rarely. These results suggest that invasion represents a shared strategy for persisting in this harsh environment, not necessarily a specific virulence trait.

Many bacterial factors have been shown to be involved in invasion, including the type 1 pili tip adhesin FimH, a key urovirulence factor in mice and cell lines (*5*, *11*–*13*, *33*). Others have suggested that FimH-independent adherence and invasion mechanisms are likely to occur, supported by the increasing list of prominent factors and the tight coordination of their expression (*34*–*37*). Here, we were able to observe invasion of umbrella cells in the absence of functional FimH, including with lower bacterial inoculum than the ones commonly used in 3D urothelial models, mice or nonhuman primate bladders (*12*, *13*, *22*, *38*–*41*). Together with the previous studies, our data reinforce the importance of developing alternative drugs that target multiple virulence factors to supplement promising anti-FimH therapeutics (*14*, *15*). In contrast, no IBC formation was observed in the absence of FimH, stressing its importance in intracellular bacteria-bacteria interactions and agreeing with the crucial role of type 1 pili in both IBC initiation and maturation in mouse bladders (*13*). Nevertheless, it is known that under static conditions, as used here, FimH binds to mannosylated glycoproteins with weak to moderate affinity (*42*), including to uroplakin Ia in umbrella cells (also expressed in our model), and only assumes a catch-bond state with high affinity during flow and shear stress. Although flowing conditions are intermittent in the bladder, more research is needed to understand which and how the other attachment/invasion coplayers cope with this condition.

Further mechanistic studies with phenotypically distinct strains of invasive bacteria species are also required to understand invasion mechanisms in human cells, as well as the stages that follow invasion, namely, intracellular growth and dispersal, access to the cytoplasm, IBC formation or expulsion, and escape from cellular pathways that are designed to prevent intracellular pathogens (e.g., autophagy). Different pathways may include host fusiform vesicle subversion (*43*), highjacking of endocytic machinery and secretory lysosomes (*44*), zipper-like mechanisms (*33*, *34*, *45*), and/or damaging of the upper cells, which, in turn, result in easier intratissue access (*11*, *45*–*47*).

Invasion has also been reported for most common non-UPEC uropathogens in mice or cell lines (*28*, *48*–*50*). In 3D-UHU, invasion was prominent with KP, which formed IBCs similar to those of UPEC, and present rarely in other species. In addition, host membrane spike-like protrusions surrounded individual KP on the surface, resembling internalization pathways described for *Salmonella* or *Shigella* (*51*), which confer high invasion efficiency even with the weaker adhesion/colonization efficiency shown here and by Rosen *et al.* (*48*, *52*). For PM, the efficient infiltration both paracellularly and intracellularly supports previous findings in cell lines and mice, for the bladder and kidney (*53*–*55*). We showed that this can occur in deeper intermediate cells, which is difficult to show in thinner mouse urothelia. However, as previously suggested (*55*, *56*), invasion was not the dominant PM lifestyle, and nor for PA, where extensive biofilm clusters were formed with mineral deposition and/or cell debris. These aggregates resembled initial stages of crystalline biofilms and encrustations, commonly detected in catheter-associated UTI animal models resulting from ureolytic biomineralization (*55*, *57*), but further investigation is needed to access their formation and nature here, in the absence of indwelling devices or neutrophil recruitment/extracellular traps.

On the other hand, some UPEC, SA, and PA could not so efficiently invade the urothelium (and/or replicate/persist). In these cases, isolated bacteria might be trapped in intracellular compartments, being easier targets for urothelial expulsion via exocytosis in exosome-like structures (*58*, *59*). In addition to the previously reported LC3 and Rab27b decorating these “cages,” we showed that Rab27a can also colocalize with expulsed bacteria. Rab27a is still not widely studied in the infection context, with moderate to absent expression reported in uninfected urothelium from different species (*60*–*62*). Here, its distinct labeling pattern (from uniform to porous) suggests a bacterial escape mechanism either via elongation, or cage degradation, as for a recent study showing that intracellular UPEC sense engulfment in fusiform vesicles, triggering expression of enzymes to escape and form IBCs (*43*).

When more subtle host defenses fail, the expulsion of bacteria in host cell fragments or wholesale cell exfoliation can serve as “last resort” measure for highly invasive (*10*) and/or cytotoxic UPEC strains (*63*). This agrees not only with the particularly marked response we observed with CFT073, but also with PM and PA, accompanied by barrier disruption and extensive cell membrane ruffling. However, this strategy may backfire for the host, as it allows establishment of deeper reservoirs, paracellular translocation, and/or cell death (as commonly observed here with PM); hence, bacteria may boost this response deliberately (*54*, *63*–*65*). We observed that most bacteria took advantage of the exfoliation process to adhere underneath cells, inserting between the upper layers. For PA, this strategy contributed to the incorporation of cell debris, possibly to thicken their protective biofilm aggregates, which was previously shown in distinct infection contexts for a few pathogens (*66*, *67*). Moreover, the “highjacking” behavior may provide temporary shelter from luminal defenses, while exploiting the sudden exposure of host adhesive molecules or matrix components. This may help to explain how poorly adherent and avirulent nonuropathogenic strains, e.g., HM50, can persist and thrive in the urothelium despite flow (*68*, *69*). Up to now, the formation of biofilms, associated with lack of host defense activation, has been the key hypothesis for the persistence of ASB strains such as HM50 (*17*, *69*). In addition, our data support the recently reported “rolling-shedding” colonization mechanism, in which UPEC was shown to hijack exfoliated cells to spread and persist throughout the urothelial surface (*70*). Moreover, host cells in poor physiological status are also easier targets for toxins and siderophores, providing a useful source of nutrients, otherwise limited in urine (*4*).

Chaining was also extremely common for most bacteria, but elongation/filamentation was mainly observed for UPEC, non-UPEC, and KP. Although filaments have been studied in the context of UPEC IBC eruption and immunity evasion (*71*, *72*), our data suggest that they are also important for dispersal and survival in the harsh human bladder microenvironment and not solely associated with pathogenicity, which is supported by very recent observations using PD07i-immortalized human cells (*73*). These bacteria also showed other alterations associated with growth in stressful environments, such as membrane vesiculation, either by active production or lysis, and nucleoid enlargement (*74*, *75*).

In our model, more aggressive strains induced the production of several proinflammatory cytokines, reminiscent of observations in mouse bladders and patients with UTI (*76*). The opposite was observed for non-UPEC, with the poorly adherent COM3 and HM50 eliciting the lowest levels. Our previous data showed that CFT073 and UTI89, at a much lower MOI of 10, also had a significant impact on 3D-UHU permeability, while UTI89 induced a stronger cytokine production compared with EF, after a longer end point of 24 hours (*25*). Here, we observed that not only these UPEC but also PA and PM had a strong impact in the model’s barrier permeability, and, in particular, they were highly cytotoxic. These highly cytotoxic strains were also the ones that induced enhanced levels of IL-1β, which agrees with previous studies where UPEC induces production of this cytokine through activation of the host inflammasome in a hemolysin-dependent manner (*77*–*79*). This enhancement was not necessarily related to invasion efficiency, as PA and CFT073 Δ*fimH* induced higher levels compared with more invasive strains. Other toxins most likely contribute to the response observed with PM and PA. For instance, in non-UTI contexts, PA pore-forming toxins also induced inflammasome activation pathways (*80*). In addition, cumulative effects of IL-1β inflammasome-independent production pathways, not addressed here, might also occur (*77*). In contrast, the nonhemolytic SA used here induced lower levels of IL-1β compared with hemolytic-positive strains (*78*, *81*), and it had the weakest impact on tissue integrity. Previously, we observed a similar effect with a different SA strain, from an asymptomatic healthy female, when compared to UPEC and EF at the lower MOI of 10 (*25*). The down-regulation of major cytokines after PM or PA infection (namely, IL-6) was notable, considering the extensive damage and cytotoxicity caused. Mechanisms associated with timing of infection or specificity in host immunity might underly these particular profiles. In addition, bacteria-induced immunomodulation (e.g., via outer membrane vesicles) is also possible (*82*) but still largely unexplored in the UTI context.

Together, the 3D-UHU microtissue model, as a good facsimile of the human urothelial microenvironment, revealed a wide variety of bacterial lifestyles and host responses, which highlights the need to develop novel therapeutics counteracting the current “one-size-fits-all” UTI treatment approach. Strategies such as filamentation, invasion, and host hijacking may aid persistence of both uropathogens and commensals but are not necessarily always correlated with virulence, reinforcing the need for more studies of the urobiome in complex contexts. For instance, more research is needed to determine evolutionary aspects and coexistence in the urothelium of isolates with intermediate phenotypes (e.g., COM1, recovered from a patient with no previous UTI history) and other strains/species. As seen for UPEC, strain-specific phenotypes will be a feature in other uropathogens as well, and their behavior in a human microenvironment should also be explored in further studies.

Nevertheless, key limitations to our model include a lack of aspects such as micturition, biomechanical stretch, and secreted factors from the kidneys and immune cells, all of which will play a role during infection. While these limitations can be addressed in future versions of this model to allow a better comparison between the different systems, 3D-UHU remains useful for studying a number of host/pathogen interactions at a key interface—the apical urothelium—in a highly controlled environment where individual variables can be tested alone or in combination. 3D-UHU should therefore prove a tractable and robust complement to animal studies that can be fine-tuned to better understand human UTI or used as a powerful platform for drug development, while providing details at microtissue, cellular, and molecular levels. This model may also serve as inspiration for the development of new cell-based platforms to study the role of the human microenvironment on infection in a variety of other organs and tissue niches.

## MATERIALS AND METHODS

### Study design

The initial goal of this study was to characterize critical events during uropathogenesis and host responses in a human-like urothelial microenvironment, with diverse UPEC clinical isolates and nonpathogenic bacteria (both commensals and an ASB strain). We hypothesized that invasion and intracellular communities could occur in different ways depending on the strain. We also wanted to know whether FimH, the main UPEC adhesin used as potential therapeutic target, demonstrates a critical role in a human tissue-like scenario. Using our innovative human microtissue urothelial model comprising barrier function, stratification/differentiation, innate immune response, and urine tolerance for long periods, we infected the bacteria during different periods 0 to 48 hours and at distinct MOI (10 to 100). Activity of the bacterial type 1 fimbriae was verified by hemagglutination assays. At fixed time points, we observed the progression of infection/colonization using confocal and scanning electron microscopy (SEM). Images were representative of a minimum of four independent biological replicates per strain. We also performed antibiotic protection assays to determine the bacterial invasion efficiency intracellularly. We investigated bacterial morphological alterations and quantified the rod-to-filament ratio after infection in a blind automated manner. To assess the effect of the bacterial strategies in the host, we determined the induced cytotoxicity by lactate dehydrogenase (LDH) release from the microtissue and its barrier permeability over time using a fluorescein isothiocyanate (FITC)–dextran (4 kDa) diffusion assay. Using microscopy, we addressed the expulsion of bacteria in exosome-like structures, host vesiculation/blebbing, and exfoliation, while the apical milieu was used to quantify cyto/chemokines production and exfoliated cells. All clinical bacterial isolates mentioned were collected for previously studies with the appropriate ethical approval in place. Given the diversity of urothelium–*E. coli* interaction, we used a similar approach to assessing infection strategies by other common Gram-positive/-negative uropathogens and respective host responses.

### Bladder human cells

Spontaneously immortalized, nontransformed human bladder epithelial cells (HBLAK; CELLnTEC) were supplied containing approximately 5 × 10^5^ cells per vial and were kept in liquid nitrogen until further use. Briefly, cells were maintained in prewarmed CnT-Prime medium (CELLnTEC), in humidified environment at 37°C and 5% CO_2_ before passaging. Passages were performed using Accutase (Sigma-Aldrich) for detachment of 80 to 90% confluent cells, incubated for 7 min at 37°C, after washing with Ca^2+^ and Mg^2+^-free phosphate-buffered saline (PBS; Gibco).

### Bacterial strains

The *E. coli* strains 83972 (HM50, ASB) and CFT073, PA PAO1 (BAA-47), SA G19 (13813), and PM 7570 (51286) were obtained from the American Type Culture Collection. KP TOP52 and UPEC UTI89, recovered from patients with acute cystitis, were provided by S. Hultgren (Washington University, St. Louis, USA), while *E. coli* MG1655 by M. El Karoui (University of Edinburgh). The MLST ST131 cystitis isolates EC6 and EC10 (*83*) were obtained from Pfizer (Department of Vaccine Design, Immunology, and Anti-Infectives Pearl River, NY, USA). Gene knockout mutants of *fimH* were generated at Pfizer from CFT073 and EC10 parental strains using the λ-red–mediated homologous recombination system as previously described (*84*). The primer pair used to verify deletion of *fimH* in the UPEC strains included the sense primer (catcggcctggcatgatgttgc) and the antisense primer (ggtactggcgacggctgc), which map to the proximal and distal *fimG* and *gntP* genes, respectively. They amplify a 0.755-kb fragment in the deletion mutants compared with a 1.658-kb fragment in the parental strains. Sanger sequencing of these fragments confirmed loss of the 0.899-kb *fimH* open-reading frame and presence of a residual scar sequence resulting from excision of the CAT cassette by the FLP recombinase. Clinical isolates from Royal Free Hospital, UK were EF (EF36), a previously reported clinical isolate from a patient with chronic UTI (*28*); UPEC ClinA, isolated from a patient with chronic UTI, and five commensal *E. coli* isolates (COM1 to COM5) recovered from clean-catch midstream urine samples of healthy individuals, as reported previously (*85*). Genotyping details about the *E. coli* strains can be found in table S1. All strains were kept in glycerol at −80°C until further use. They were generally maintained at 37°C under static conditions in optimal medium before experiments: Luria broth for *E. coli* (LB; Sigma-Aldrich); tryptone soya broth (Oxoid) for PA, SA, PM, and EF; and nutrient broth (Oxoid) for KP.

### Generation of 3D human urothelial model

3D-UHU was generated as previously described (*25*). Briefly, between passages 8 to 12, 80 to 90% confluent cells were detached as above described and 3 × 10^5^ cells/ml in prewarmed CnT-Prime were seeded onto 12-mm, 0.4-μm pore polycarbonate filter membranes in plastic inserts standing in 12-well Transwell plates (Corning), while the basal chamber was filled with 1.5 ml of the same medium. After 2 days of incubation at 37°C and 5% CO_2_, medium in both apical and basal chambers was replaced by calcium-rich (1.2 mM) differentiation barrier medium (CnT-Prime-3D medium, CELLnTEC), designated day 0. After overnight incubation, medium in the apical chambers was replaced with commercially available filter-sterilized human urine pooled from 10 individuals, both sexes (BioIVT), while fresh medium was replaced in the basal chamber. Urine/3D medium changes every 3 days were performed until days 18 to 20, when models were used for subsequent experiments.

### Bacterial growth curves

Growth of bacteria was monitored by optical density (OD) measurements at 600 nm over 24 hours under 37°C in LB medium, 50% urine diluted in CnT-Prime-3D medium, or 100% human urine (initial inoculum OD_600nm_, 0.006 ± 0.003), using a microtiter plate reader Tecan Spark.

### Bacterial inoculations

Before infection, bacterial strains were grown statically overnight from frozen stocks at 37°C and then subcultured in fresh optimal medium. *E. coli* was then grown for 48 hours statically at 37°C, while one overnight incubation was used for the other species of bacteria. Bacterial numbers were quantified using QUANTOM Tx Microbial Cell Counter (Logos), according to the manufacturer’s instructions. For infection, bacteria were inoculated in the apical chamber using MOI of 10 to 100 in urine, depending on the experiment. For reproducibility purposes, MOI was calculated on the basis of approximately 30,000 umbrella cells on the mature microtissue apical surface, as estimated by surface area and cell size. CnT-Prime-3D medium was replaced in the basal chamber.

### Hemagglutination assays

Bacteria were grown in LB broth statically as described above and then diluted to an OD_600nm_ of 0.5. Cells were harvested by centrifugation at 4000*g* for 5 min and resuspended in PBS or in 1% mannose (to inhibit type I fimbriae binding). Suspensions were then mixed with 5% or 1% (v/v) of guinea pig erythrocytes (Rockland). Hemagglutination was visually monitored over 0 to 4 hours of incubation at 4°C in microtiter wells or imaged in glass slides after 30 min incubation at room temperature, using a Leica inverted microscope DMi1 (Leica Microsystems).

### Biofilm quantification assay

After growth as aforementioned, the formation of biofilms from all bacterial cultures was assessed in LB and 25% urine. Bacterial suspensions were adjusted to mimic an MOI of 15 and 30, onto Calgary biofilm devices (Innovotech), which are microtiter 96-well plates with lids that have pegs extending into each well. After static incubation at 37°C and 5% CO_2_ for 24 hours, the lids were removed and air dried for 20 min. The pegs were then submerged into a new plate containing 200 μl of crystal violet stain and incubated at room temperature for 30 min. Subsequently, the pegs were gently rinsed with distilled water and air-dried before being submerged in 200 μl of 33% acetic acid (Sigma-Aldrich) for 15 min. Absorbance of the dissolved biofilms was measured using a Tecan Spark microplate reader at OD_550nm_.

### FITC-dextran barrier permeability assay

To monitor the 3D-UHU barrier integrity upon infection, bacteria were inoculated at an MOI of 15 in 4-kDa FITC-dextran (1 mg/ml; Sigma-Aldrich) dissolved in CnT-Prime-3D. An empty Transwell and an uninfected model served as controls. After 0, 2, 4, 6, 8, 20, 22 and 24 hpi, 50 μl of basal medium was transferred into a 96-well clear-bottom black polystyrene microplate (Corning). Fluorescence intensity was measured using a fluorescence plate reader (Tecan Spark) at 490-nm excitation and 520-nm emission values.

### LDH cytotoxicity assay

Cytotoxicity caused by the bacteria at MOI 100 after 12 hpi in 3D-UHU was assessed by quantification of the amount of LDH released into the apical milieu, using the commercially available CyQUANT LDH Cytotoxicity Assay (Thermo Fisher Scientific) as recommended by the manufacturer.

### Gentamicin protection assay

After 12 hpi at an MOI of 100 (initial inoculum of 10^6^ bacterial cells), the medium in the apical chambers was replaced by gentamicin (500 μg/ml; in 100% urine), 150 to 250× above minimum inhibitory concentration [performed as described in Wiegand *et al.* (*86*)], and in the basal chambers by fresh CnT-Prime-3D. After incubation for 8 hours at 37°C and 5% CO_2_, the total volume of medium in basal and apical chambers was spread onto LB plates for colony-forming unit counting to confirm the absence of growth after overnight incubation at 37°C. Inserts were washed twice with PBS and incubated with 1% Triton X-100 for 20 min at 37°C and 5% CO_2_, and mechanical lysis was performed by scraping the surface and pipetting up and down 10 times. Cell lysates were then serial diluted and spread onto LB agar plates for CFU counting after overnight incubation at 37°C.

### Recovery and analysis of exfoliated cells

After 3, 6, or 12 hpi, exfoliated host cells in the apical milieu were quantified in 4 and 20 μl of samples using Acella 20 and 100 (respectively) sample carriers and the fluidlab R-300 automated cell counter (Anvajo Biotech). From the remaining volume, 100 μl was cytocentrifuged at 800 rpm for 5 min onto glass slides using a Cytospin 2 centrifuge (Shandon). A hydrophobic pen was used to define the location of cytospun cells for subsequent sample processing. Slides were washed with PBS and fixed overnight with 4% paraformaldehyde (PFA) in PBS (Invitrogen) at 4°C. The following day, PFA was replaced by 1× PBS, and inserts were kept at 4°C until staining.

### Immunofluorescence staining and microscopy

All 3D-UHU cultures on membranes and cytospun shed cells were washed three times with PBS to remove loosely adherent bacteria before fixation in 4% PFA in PBS. For the detection of umbrella cell biomarkers, fixed uninfected membranes were washed twice with 1× Hank’s balanced salt solution (Gibco) and stained with wheat germ agglutinin (5 μg/ml) conjugated to Alexa Fluor 555 (Invitrogen) in Hank’s balanced salt solution for 2 hours at room temperature in the dark. Membranes were then washed with 1× Ca^2+^- and Mg^2+^-free PBS before blocking with 5% normal goat serum (NGS; Thermo Fisher Scientific) in 1% PBS/bovine serum albumin (BSA; Sigma-Aldrich) for 1 hour. Subsequently, membranes were incubated overnight at 4°C with primary antibodies diluted in 1% NGS in 1% BSA/PBS at 1:50 for rabbit anti–cytokeratin-20 polyclonal antibody (Invitrogen), mouse anti–uroplakin Ia monoclonal antibody (Thermo Fisher Scientific), or 1:30 for mouse anti–uroplakin III monoclonal antibody (Invitrogen). After being washed three times in 1% BSA/PBS, membranes were then incubated for 2 hours at room temperature with a 1:200 dilution of the respective secondary antibody: goat anti-mouse or goat anti-rabbit conjugated to Alexa Fluor 488 (Invitrogen). Membranes were then washed three times in 1% BSA/PBS, and labeling of F-actin was performed with 1:500 dilution of Alexa Fluor 488–conjugated Phalloidin (Invitrogen) for 1 hour at room temperature after permeabilization with 0.2% Triton X-100 (Sigma-Aldrich) in PBS for 35 min at room temperature, when wheat germ agglutinin was not previously used. DNA were stained with 4′,6-diamidino-2-phenylindole (DAPI; 1 μg/ml; Invitrogen) for 15 min at room temperature, and membranes were mounted with ProLong Glass Antifade Mountant (Invitrogen) onto glass slides for imaging. For infected membranes and slides with cytospun exfoliated cells, bacteria were labeled with a 1:50 dilution in PBS of either the following primary antibodies, at 4°C overnight: mouse anti-O6 (for CFT073), human anti-O25b (for EC10 and mutant), chicken anti-PA (Abcam), rabbit anti-KP (Invitrogen) and rabbit anti-PM (Invitrogen), rabbit anti-LC3A/B (Cell Signaling Technology), rabbit anti-Rab27a (Invitrogen), or mouse anti–uroplakin IIIa (Santa Cruz Biotechnology); or with FITC-conjugated polyclonal antibodies (Invitrogen) at room temperature for 3 hours: anti-*Streptococcus* group B, anti–*E. coli* serotype O/K, or anti–*Enterococcus* sp. Primary antibody incubations were followed by a second incubation with a 1:300 dilution in 1% NGS of goat anti-chicken, anti-rabbit, anti-mouse, or anti-human secondary antibodies conjugated with Alexa Fluor 488 (Invitrogen), respectively, for 2 hours at room temperature. Wheat germ agglutinin or Phalloidin, DAPI staining, and mounting of the samples were performed as aforementioned.

To examine intracellular bacteria by confocal microscopy, the above staining strategies allowed differential color detection, with extracellular bacteria stained with antibacteria FITC-conjugated antibody (green/yellow) and DAPI (blue), whereas intracellular bacteria were only stained with blue, and could be distinguished from host cell nuclei by morphology. IBCs or intracellular isolated bacteria were identified after 3D rendering and analysis of the images. As a control of the fixation procedure above, with and without subsequent membrane permeabilization, infected/uninfected microtissues were stained with a mouse monoclonal antimitochondria antibody Cy3 conjugate (Merck) incubated overnight at 4°C, to confirm that without permeabilization, there was no access of antibodies into the microtissue.

Visualization of stained organoids was performed by confocal laser scanning microscopy using a Leica SP8 microscope, with inspection for intracellular bacteria in top-down views and *Z*-stacks (side views). Images were processed using the Leica Application Suite (LASX), Advanced Fluorescence 3.1.0 build 8587 software.

### Scanning electron microscopy

Fixation of samples for SEM was performed in 2.5% glutaraldehyde/2% PFA in 0.1 M sodium cacodylate (CAC; TAAB Laboratories) buffer for 30 min at room temperature. Fixed membranes were then incubated with 1% osmium tetroxide (TAAB Laboratories)/1.5% potassium ferricyanide (Sigma-Aldrich) for 1 hour at 4°C, followed by three washes with 0.1 M CAC buffer. Subsequently, they were incubated in 1% tannic acid (TAAB Laboratories) in 0.05 M CAC buffer in the dark at room temperature for 40 min, followed by two washes with 0.05 M CAC buffer and one wash with distilled and deionized water (ddH_2_O). Membranes were then dehydrated in graded ethanol (Sigma-Aldrich) series: 2 min of incubation each in 50, 70, and 90% and 10 min in 100% ethanol, twice. Dehydrated microtissues were completely dried with a Leica EM CPD300 critical point dryer. The membranes were then detached from the plastic inserts using a scalpel and were mounted onto aluminum stubs using carbon sticky tabs, where membranes were sputter-coated with 10 nm of gold. SEM images were acquired using a Zeiss Gemini 300 with a working distance of 8-mm, 1.5-kV accelerating voltage using secondary electron (SE2) detector and processed with Zeiss Atlas 5 software. False coloring of the images was performed using GIMP 2.10.

### Cyto/chemokines profiling

The apical milieu from infections at MOI 100 after 12 hours was centrifuged at 10,000*g* for 5 min, followed by another centrifugation at 13,000*g* for 8 min, to remove cellular/bacterial debris. Supernatants were used to quantify the amount of cyto/chemokines produced by the urothelial microtissues before and after infection using a human premixed multianalyte customized kit in a Luminex bead–based immunoassay (R&D Systems) according to the manufacturer’s instructions. The final suspensions in wash buffer were analyzed using a Luminex 200 machine, for the analysis of the following 16 analytes: CCL5 (RANTES), CXCL1 (GRO-α), CXCL2 (GRO-β), GM-CSF (CSF2), G-CSF (CSF3), interferon-γ, tumor necrosis factor–α, IL-1α, IL1-β, IL-6, CXCL8 (IL-8), IL-18, Lactoferrin, Lipocalin-2 (NGAL), Pentraxin 3, and osteopontin.

### Statistical analysis

Data were expressed as means ± SD, plotted, and analyzed using GraphPad Prism version 9.3.1. Statistical significance between replicates was determined using analysis of variance (ANOVA), followed by Bonferroni’s test.

For quantification of PA and *E. coli* rod and filament lengths, confocal images were converted in 16-bit images and analyzed in the ImageJ software using the MicrobeJ plugin (*87*). A total of 1000 bacteria were counted across biological triplicates per strain, using three to five microtissue regions per replicate, with the exception of COM3, where 15 regions were needed to achieve equivalent total bacterial count. Bacterial lengths were gated from 0 to 4 μm for rods and >4 μm for filaments, while declustering was manually performed. Filaments longer than the area of view or overlapping in a way that could not be declustered were discarded from quantification.

## References

[1] F. M. E. Wagenlehner, T. E. Bjerklund Johansen, T. Cai, B. Koves, J. Kranz, A. Pilatz, Z. Tandogdu, Epidemiology, definition and treatment of complicated urinary tract infections. Nat. Rev. Urol. 17, 586–600 (2020). 32843751 10.1038/s41585-020-0362-4

[2] World Health Organization, *Global Report on Infection Prevention and Control* (World Health Organization, 2022).

[3] Antimicrobial Resistance Collaborators, Global burden of bacterial antimicrobial resistance in 2019: A systematic analysis. Lancet 399, 629–655 (2022). 35065702 10.1016/S0140-6736(21)02724-0 PMC8841637

[4] A. L. Flores-Mireles, J. N. Walker, M. Caparon, S. J. Hultgren, Urinary tract infections: Epidemiology, mechanisms of infection and treatment options. Nat. Rev. Microbiol. 13, 269–284 (2015). 25853778 10.1038/nrmicro3432 PMC4457377

[5] D. A. Hunstad, S. S. Justice, Intracellular lifestyles and immune evasion strategies of uropathogenic *Escherichia coli*. Annu. Rev. Microbiol. 64, 203–221 (2010). 20825346 10.1146/annurev.micro.112408.134258

[6] N. J. De Nisco, M. Neugent, J. Mull, L. Chen, A. Kuprasertkul, M. De, S. Santos, K. L. Palmer, P. Zimmern, K. Orth, Direct detection of tissue-resident bacteria and chronic inflammation in the bladder wall of postmenopausal women with recurrent urinary tract infection. J. Mol. Biol. 431, 4368–4379 (2019). 31002774 10.1016/j.jmb.2019.04.008 PMC6801050

[7] D. A. Rosen, T. M. Hooton, W. E. Stamm, P. A. Humphrey, S. J. Hultgren, S. M. Opal, Detection of intracellular bacterial communities in human urinary tract infection. PLOS Med. 4, e329 (2007). 18092884 10.1371/journal.pmed.0040329 PMC2140087

[8] L. Robino, P. Scavone, L. Araujo, G. Algorta, P. Zunino, R. Vignoli, Detection of intracellular bacterial communities in a child with *Escherichia coli* recurrent urinary tract infections. Pathog. Dis. 68, 78–81 (2013). 23733378 10.1111/2049-632X.12047

[9] L. Robino, P. Scavone, L. Araujo, G. Algorta, P. Zunino, M. Catalina Pírez, R. Vignoli, Intracellular bacteria in the pathogenesis of *Escherichia coli* urinary tract infection in children. Clin. Infect. Dis. 59, e158–e164 (2014). 25091303 10.1093/cid/ciu634 PMC4650771

[10] Y. Cheng, Z. Chen, J. A. Gawthorne, C. Mukerjee, K. Varettas, K. J. Mansfield, M. A. Schembri, K. H. Moore, Detection of intracellular bacteria in exfoliated urothelial cells from women with urge incontinence. Pathog. Dis. 74, ftw67 (2016). 10.1093/femspd/ftw067 27402784

[11] M. A. Mulvey, J. D. Schilling, S. J. Hultgren, Establishment of a persistent *Escherichia coli* reservoir during the acute phase of a bladder infection. Infect. Immun. 69, 4572–4579 (2001). 11402001 10.1128/IAI.69.7.4572-4579.2001 PMC98534

[12] F. K. Bahrani-Mougeot, E. L. Buckles, C. V. Lockatell, J. R. Hebel, D. E. Johnson, C. M. Tang, M. S. Donnenberg, Type 1 fimbriae and extracellular polysaccharides are preeminent uropathogenic *Escherichia coli* virulence determinants in the murine urinary tract. Mol. Microbiol. 45, 1079–1093 (2002). 12180926 10.1046/j.1365-2958.2002.03078.x

[13] K. J. Wright, P. C. Seed, S. J. Hultgren, Development of intracellular bacterial communities of uropathogenic *Escherichia coli* depends on type 1 pili. Cell. Microbiol. 9, 2230–2241 (2007). 17490405 10.1111/j.1462-5822.2007.00952.x

[14] M. Sarshar, P. Behzadi, C. Ambrosi, C. Zagaglia, A. T. Palamara, D. Scribano, FimH and anti-adhesive therapeutics: A disarming strategy against uropathogens. Antibiotics 9, 397 (2020). 32664222 10.3390/antibiotics9070397 PMC7400442

[15] N. Foroogh, M. Rezvan, K. Ahmad, S. Mahmood, Structural and functional characterization of the FimH adhesin of uropathogenic *Escherichia coli* and its novel applications. Microb. Pathog. 161, 105288 (2021). 34780972 10.1016/j.micpath.2021.105288

[16] V. Roos, G. C. Ulett, M. A. Schembri, P. Klemm, The asymptomatic bacteriuria *Escherichia coli* strain 83972 outcompetes uropathogenic *E. coli* strains in human urine. Infect. Immun. 74, 615–624 (2006). 16369018 10.1128/IAI.74.1.615-624.2006 PMC1346649

[17] P. Klemm, V. Hancock, M. A. Schembri, Mellowing out: Adaptation to commensalism by *Escherichia coli* asymptomatic bacteriuria strain 83972. Infect. Immun. 75, 3688–3695 (2007). 17502385 10.1128/IAI.01730-06 PMC1951988

[18] R. E. Watts, M. Totsika, V. L. Challinor, A. N. Mabbett, G. C. Ulett, J. J. D. Voss, M. A. Schembri, Contribution of siderophore systems to growth and urinary tract colonization of asymptomatic bacteriuria *Escherichia coli*. Infect. Immun. 80, 333–344 (2012). 21930757 10.1128/IAI.05594-11 PMC3255690

[19] E. Salvador, F. Wagenlehner, C. D. Köhler, A. Mellmann, J. Hacker, C. Svanborg, U. Dobrindt, Comparison of asymptomatic bacteriuria *Escherichia coli* isolates from healthy individuals versus those from hospital patients shows that long-term bladder colonization selects for attenuated virulence phenotypes. Infect. Immun. 80, 668–678 (2012). 22104113 10.1128/IAI.06191-11 PMC3264318

[20] A. R. Eberly, C. J. Beebout, C. Man, C. Tong, G. T. Van Horn, H. D. Green, M. J. Fitzgerald, S. De, E. K. Apple, A. C. Schrimpe-Rutledge, S. G. Codreanu, S. D. Sherrod, J. A. Mclean, D. B. Clayton, C. W. Stratton, J. E. Schmitz, M. Hadjifrangiskou, Defining a molecular signature for uropathogenic versus urocolonizing *Escherichia coli*: The status of the field and new clinical opportunities. J. Mol. Biol. 432, 786–804 (2020). 31794727 10.1016/j.jmb.2019.11.008 PMC7293133

[21] B. O. Murray, C. Flores, C. Williams, D. A. Flusberg, E. E. Marr, K. M. Kwiatkowska, J. L. Charest, B. C. Isenberg, J. L. Rohn, Recurrent urinary tract infection: A mystery in search of better model systems. Front. Cell. Infect. Microbiol. 11, 691210 (2021). 34123879 10.3389/fcimb.2021.691210 PMC8188986

[22] Y. C. Smith, K. K. Grande, S. B. Rasmussen, A. D. O’Brien, Novel three-dimensional organoid model for evaluation of the interaction of uropathogenic Escherichia coli with terminally differentiated human urothelial cells. Infect. Immun. 74, 750–757 (2006). 16369034 10.1128/IAI.74.1.750-757.2006 PMC1346604

[23] S. C. Baker, S. Shabir, J. Southgate, Biomimetic urothelial tissue models for the in vitro evaluation of barrier physiology and bladder drug efficacy. Mol. Pharm. 11, 1964–1970 (2014). 24697150 10.1021/mp500065m

[24] K. Sharma, V. V. Thacker, N. Dhar, M. Clapés Cabrer, A. Dubois, F. Signorino-Gelo, J. Mullenders, G. W. Knott, H. Clevers, J. D. McKinney, Early invasion of the bladder wall by solitary bacteria protects UPEC from antibiotics and neutrophil swarms in an organoid model. Cell Rep. 36, 109351 (2021). 34289360 10.1016/j.celrep.2021.109351

[25] N. V. Jafari, J. L. Rohn, An immunoresponsive three-dimensional urine-tolerant human urothelial model (3D-UHU) to study urinary tract infection. Front. Cell. Infect. Microbiol. 13, 269 (2023). 10.3389/fcimb.2023.1128132 PMC10083561 37051302

[26] N. V. Jafari, J. L. Rohn, The urothelium: A multi-faceted barrier against a harsh environment. Mucosal Immunol. 15, 1127–1142 (2022). 36180582 10.1038/s41385-022-00565-0 PMC9705259

[27] G. G. Anderson, J. J. Palermo, J. D. Schilling, R. Roth, J. Heuser, S. J. Hultgren, Intracellular bacterial biofilm-like pods in urinary tract infections. Science 301, 105–107 (2003). 12843396 10.1126/science.1084550

[28] H. Horsley, D. Dharmasena, J. Malone-Lee, J. L. Rohn, A urine-dependent human urothelial organoid offers a potential alternative to rodent models of infection. Sci. Rep. 8, 1238 (2018). 29352171 10.1038/s41598-018-19690-7 PMC5775255

[29] L. Reitzer, P. Zimmern, Rapid growth and metabolism of uropathogenic *Escherichia coli* in relation to urine composition. Clin. Microbiol. Rev. 33, e00101-19 (2020). 10.1128/CMR.00101-19 PMC6927312 31619395

[30] R. M. Vejborg, P. Klemm, Cellular chain formation in *Escherichia coli* biofilms. Microbiology 155, 1407–1417 (2009). 19383712 10.1099/mic.0.026419-0

[31] S. Paudel, K. Bagale, S. Patel, N. J. Kooyers, R. Kulkarni, Human urine alters methicillin-resistant staphylococcus aureus virulence and transcriptome. Appl. Environ. Microbiol. 87, e0074421 (2021). 34105987 10.1128/AEM.00744-21 PMC8315183

[32] M. Castillo-Martin, J. Domingo-Domenech, O. Karni-Schmidt, T. Matos, C. Cordon-Cardo, Molecular pathways of urothelial development and bladder tumorigenesis. Urol. Oncol. Semin. Orig. Investig. 28, 401–408 (2010). 10.1016/j.urolonc.2009.04.019 20610278

[33] J. J. Martinez, S. J. Hultgren, Requirement of rho-family GTPases in the invasion of type 1-piliated uropathogenic *Escherichia coli*. Cell. Microbiol. 4, 19–28 (2002). 11856170 10.1046/j.1462-5822.2002.00166.x

[34] I. Kansau, C. Berger, M. Hospital, R. Amsellem, V. Nicolas, A. L. Servin, M. F. Bernet-Camard, Zipper-like internalization of Dr-positive *Escherichia coli* by epithelial cells is preceded by an adhesin-induced mobilization of raft-associated molecules in the initial step of adhesion. Infect. Immun. 72, 3733–3742 (2004). 15213113 10.1128/IAI.72.7.3733-3742.2004 PMC427432

[35] B. K. Dhakal, R. R. Kulesus, M. A. Mulvey, Mechanisms and consequences of bladder cell invasion by uropathogenic *Escherichia coli*. Eur. J. Clin. Invest. 38, 2–11 (2008). 18616559 10.1111/j.1365-2362.2008.01986.x

[36] R. E. Berry, D. J. Klumpp, A. J. Schaeffer, Urothelial cultures support intracellular bacterial community formation by uropathogenic *Escherichia coli*. Infect. Immun. 77, 2762–2772 (2009). 19451249 10.1128/IAI.00323-09 PMC2708588

[37] A. J. Lewis, A. C. Richards, M. A. Mulvey, Invasion of host cells and tissues by uropathogenic bacteria. Urin. Tract Infect. Mol. Pathog. Clin. Manag. 4, 359–381 (2016). 10.1128/microbiolspec.UTI-0026-2016 PMC5244466 28087946

[38] S. Langermann, S. Palaszynski, M. Barnhart, G. Auguste, J. S. Pinkner, J. Burlein, P. Barren, S. Koenig, S. Leath, C. H. Jones, S. J. Hultgren, Prevention of mucosal *Escherichia coli* infection by FimH-adhesin-based systemic vaccination. Science 276, 607–611 (1997). 9110982 10.1126/science.276.5312.607

[39] S. Langermann, R. Möllby, J. E. Burlein, S. R. Palaszynski, C. G. Auguste, A. DeFusco, R. Strouse, M. A. Schenerman, S. J. Hultgren, J. S. Pinkner, J. Winberg, L. Guldevall, M. Söderhäll, K. Ishikawa, S. Normark, S. Koenig, Vaccination with FimH adhesin protects cynomolgus monkeys from colonization and infection by uropathogenic *Escherichia coli*. J. Infect. Dis. 181, 774–778 (2000). 10669375 10.1086/315258

[40] M. A. Mulvey, Y. S. Lopez-Boado, C. L. Wilson, R. Roth, W. C. Parks, J. Heuser, S. J. Hultgren, Induction and evasion of host defenses by type 1-piliated uropathogenic *Escherichia coli*. Science 282, 1494–1497 (1998). 9822381 10.1126/science.282.5393.1494

[41] S. L. Chen, C. S. Hung, J. S. Pinkner, J. N. Walker, C. K. Cusumano, Z. Li, J. Bouckaert, J. I. Gordon, S. J. Hultgren, Positive selection identifies an in vivo role for FimH during urinary tract infection in addition to mannose binding. Proc. Natl. Acad. Sci. U.S.A. 106, 22439–22444 (2009). 20018753 10.1073/pnas.0902179106 PMC2794649

[42] M. M. Sauer, R. P. Jakob, J. Eras, S. Baday, D. Eriş, G. Navarra, S. Bernèche, B. Ernst, T. Maier, R. Glockshuber, Catch-bond mechanism of the bacterial adhesin FimH. Nat. Commun. 7, 1–13 (2016). 10.1038/ncomms10738 PMC4786642 26948702

[43] Y. Pang, Z. Cheng, S. Zhang, S. Li, X. Li, X. Li, X. Zhang, X. Li, Y. Feng, H. Cui, Z. Chen, L. Liu, Q. Li, J. Huang, M. Zhang, S. Zhu, L. Wang, L. Feng, Bladder epithelial cell phosphate transporter inhibition protects mice against uropathogenic *Escherichia coli* infection. Cell Rep. 39, 110698 (2022). 35443182 10.1016/j.celrep.2022.110698

[44] W. J. Kim, A. E. Shea, J. H. Kim, Y. Daaka, Uropathogenic *Escherichia coli* invades bladder epithelial cells by activating kinase networks in host cells. J. Biol. Chem. 293, 16518–16527 (2018). 30166343 10.1074/jbc.RA118.003499 PMC6200948

[45] D. S. Eto, T. A. Jones, J. L. Sundsbak, M. A. Mulvey, Integrin-mediated host cell invasion by type 1-piliated uropathogenic *Escherichia coli*. PLOS Pathog. 3, e100 (2007). 17630833 10.1371/journal.ppat.0030100 PMC1914067

[46] A. Doye, A. Mettouchi, G. Bossis, R. Clément, C. Buisson-Touati, G. Flatau, L. Gagnoux, M. Piechaczyk, P. Boquet, E. Lemichez, CNF1 exploits the ubiquitin-proteasome machinery to restrict rho GTPase activation for bacterial host cell invasion. Cell 111, 553–564 (2002). 12437928 10.1016/s0092-8674(02)01132-7

[47] K. Nagamatsu, T. J. Hannan, R. L. Guest, M. Kostakioti, M. Hadjifrangiskou, J. Binkley, K. Dodson, T. L. Raivio, S. J. Hultgren, Dysregulation of *Escherichia coli* α-hemolysin expression alters the course of acute and persistent urinary tract infection. Proc. Natl. Acad. Sci. U.S.A. 112, E871–E880 (2015). 25675528 10.1073/pnas.1500374112 PMC4345586

[48] D. A. Rosen, J. S. Pinkner, J. M. Jones, J. N. Walker, S. Clegg, S. J. Hultgren, Utilization of an intracellular bacterial community pathway in *Klebsiella pneumoniae* urinary tract infection and the effects of FimK on type 1 pilus expression. Infect. Immun. 76, 3337–3345 (2008). 18411285 10.1128/IAI.00090-08 PMC2446714

[49] S. Y. Leclercq, M. J. Sullivan, D. S. Ipe, J. P. Smith, A. W. Cripps, G. C. Ulett, Pathogenesis of Streptococcus urinary tract infection depends on bacterial strain and β-hemolysin/cytolysin that mediates cytotoxicity, cytokine synthesis, inflammation and virulence. Sci. Rep. 6, 29000 (2016). 27383371 10.1038/srep29000 PMC4935997

[50] J. N. Newman, R. V. Floyd, J. L. Fothergill, Invasion and diversity in *Pseudomonas aeruginosa* urinary tract infections. J. Med. Microbiol. 71, 001458 (2022). 35275806 10.1099/jmm.0.001458 PMC9176269

[51] A. Weiner, J. Enninga, The pathogen–host interface in three dimensions: Correlative FIB/SEM applications. Trends Microbiol. 27, 426–439 (2019). 30600140 10.1016/j.tim.2018.11.011

[52] D. A. Rosen, J. S. Pinkner, J. N. Walker, J. S. Elam, J. M. Jones, S. J. Hultgren, Molecular variations in *Klebsiella pneumoniae* and *Escherichia coli* FimH affect function and pathogenesis in the urinary tract. Infect. Immun. 76, 3346–3356 (2008). 18474655 10.1128/IAI.00340-08 PMC2446687

[53] H. L. T. Mobley, R. Belas, V. Lockatell, G. Chippendale, A. L. Trifillis, D. E. Johnson, J. W. Warren, Construction of a flagellum-negative mutant of *Proteus mirabilis*: Effect on internalization by human renal epithelial cells and virulence in a mouse model of ascending urinary tract infection. Infect. Immun. 64, 5332–5340 (1996). 8945585 10.1128/iai.64.12.5332-5340.1996 PMC174527

[54] P. Alamuri, M. Löwer, J. A. Hiss, S. D. Himpsl, G. Schneider, H. L. T. Mobley, Adhesion, invasion, and agglutination mediated by two trimeric autotransporters in the human uropathogen *Proteus mirabilis*. Infect. Immun. 78, 4882–4894 (2010). 20805336 10.1128/IAI.00718-10 PMC2976323

[55] J. N. Schaffer, A. N. Norsworthy, T. T. Sun, M. M. Pearson, *Proteus mirabilis* fimbriae- and urease-dependent clusters assemble in an extracellular niche to initiate bladder stone formation. Proc. Natl. Acad. Sci. U.S.A. 113, 4494–4499 (2016). 27044107 10.1073/pnas.1601720113 PMC4843424

[56] A. M. Jansen, V. Lockatell, D. E. Johnson, H. L. T. Mobley, Mannose-resistant *Proteus*-like fimbriae are produced by most *Proteus mirabilis* strains infecting the urinary tract, dictate the in vivo localization of bacteria, and contribute to biofilm formation. Infect. Immun. 72, 7294–7305 (2004). 15557655 10.1128/IAI.72.12.7294-7305.2004 PMC529131

[57] X. Li, H. Zhao, C. V. Lockatell, C. B. Drachenberg, D. E. Johnson, H. L. T. Mobley, Visualization of *Proteus mirabilis* within the matrix of urease-induced bladder stones during experimental urinary tract infection. Infect. Immun. 70, 389–394 (2002). 11748205 10.1128/IAI.70.1.389-394.2002 PMC127628

[58] Y. Miao, G. Li, X. Zhang, H. Xu, S. N. Abraham, A TRP channel senses lysosome neutralization by pathogens to trigger their expulsion. Cell 161, 1306–1319 (2015). 26027738 10.1016/j.cell.2015.05.009 PMC4458218

[59] C. S. Joshi, A. Mora, P. A. Felder, I. U. Mysorekar, NRF2 promotes urothelial cell response to bacterial infection by regulating reactive oxygen species and RAB27B expression. Cell Rep. 37, 109856 (2021). 34686330 10.1016/j.celrep.2021.109856

[60] Y. Chen, X. Guo, F. M. Deng, F. X. Liang, W. Sun, M. Ren, T. Izumi, D. D. Sabatini, T. T. Sun, G. Kreibich, Rab27b is associated with fusiform vesicles and may be involved in targeting uroplakins to urothelial apical membranes. Proc. Natl. Acad. Sci. U.S.A. 100, 14012–14017 (2003). 14625374 10.1073/pnas.2436350100 PMC283537

[61] J. Liu, X. Gong, X. Zhu, D. Xue, Y. Liu, P. Wang, Rab27A overexpression promotes bladder cancer proliferation and chemoresistance through regulation of NF-κB signaling. Oncotarget 8, 75272–75283 (2017). 29088864 10.18632/oncotarget.20775 PMC5650419

[62] L. I. Gallo, M. G. Dalghi, D. R. Clayton, W. G. Ruiz, P. Khandelwal, G. Apodaca, RAB27B requirement for stretch-induced exocytosis in bladder umbrella cells. Am. J. Physiol. Cell Physiol. 314, C349–C365 (2018). 29167152 10.1152/ajpcell.00218.2017 PMC6335015

[63] A. Jermy, UPEC helps host to exfoliate. Nat. Rev. Microbiol. 10, 159–159 (2012). 22330905 10.1038/nrmicro2758

[64] B. K. Dhakal, M. A. Mulvey, The UPEC pore-forming toxin α-hemolysin triggers proteolysis of host proteins to disrupt cell adhesion, inflammatory, and survival pathways. Cell Host Microbe 11, 58–69 (2012). 22264513 10.1016/j.chom.2011.12.003 PMC3266558

[65] M. G. Blango, E. M. Ott, A. Erman, P. Veranic, M. A. Mulvey, Forced resurgence and targeting of intracellular uropathogenic *Escherichia coli* reservoirs. PLOS ONE 9, e93327 (2014). 24667805 10.1371/journal.pone.0093327 PMC3965547

[66] G. W. Burnham, H. D. Cavanagh, D. M. Robertson, The impact of cellular debris on *Pseudomonas aeruginosa* adherence to silicone hydrogel contact lenses and contact lens storage cases. Eye Contact Lens 38, 7–15 (2012). 22138709 10.1097/ICL.0b013e31823bad0e PMC7384716

[67] M. T. Buhmann, P. Stiefel, K. Maniura-Weber, Q. Ren, In vitro biofilm models for device-related infections. Trends Biotechnol. 34, 945–948 (2016). 27344424 10.1016/j.tibtech.2016.05.016

[68] P. Andersson, I. Engberg, G. Lidin-Janson, K. Lincoln, R. Hull, S. Hull, C. Svanborg, Persistence of *Escherichia coli* bacteriuria is not determined by bacterial adherence. Infect. Immun. 59, 2915–2921 (1991). 1879917 10.1128/iai.59.9.2915-2921.1991 PMC258113

[69] I. Ambite, D. Butler, M. L. Y. Wan, T. Rosenblad, T. H. Tran, S. M. Chao, C. Svanborg, Molecular determinants of disease severity in urinary tract infection. Nat. Rev. Urol. 18, 468–486 (2021). 34131331 10.1038/s41585-021-00477-x PMC8204302

[70] B. Zalewska-Piątek, M. Olszewski, T. Lipniacki, S. Błoński, M. Wieczór, P. Bruździak, A. Skwarska, B. Nowicki, S. Nowicki, R. Piątek, A shear stress micromodel of urinary tract infection by the *Escherichia coli* producing Dr adhesin. PLOS Pathog. 16, e1008247 (2020). 31917805 10.1371/journal.ppat.1008247 PMC7004390

[71] S. S. Justice, D. A. Hunstad, P. C. Seed, S. J. Hultgren, Filamentation by *Escherichia coli* subverts innate defenses during urinary tract infection. Proc. Natl. Acad. Sci. U.S.A. 103, 19884–19889 (2006). 17172451 10.1073/pnas.0606329104 PMC1750882

[72] T. E. Andersen, S. Khandige, M. Madelung, J. Brewer, H. J. Kolmos, J. Møller-Jensen, *Escherichia coli* uropathogenesis in vitro: Invasion, cellular escape, and secondary infection analyzed in a human bladder cell infection model. Infect. Immun. 80, 1858–1867 (2012). 22354025 10.1128/IAI.06075-11 PMC3347433

[73] B. Söderström, M. J. Pittorino, D. O. Daley, I. G. Duggin, Assembly dynamics of FtsZ and DamX during infection-related filamentation and division in uropathogenic *E. coli*. Nat. Commun. 13, 3648 (2022). 35752634 10.1038/s41467-022-31378-1 PMC9233674

[74] M. Wainwright, L. T. Canham, K. Al-Wajeeh, C. L. Reeves, Morphological changes (including filamentation) in *Escherichia coli* grown under starvation conditions on silicon wafers and other surfaces. Lett. Appl. Microbiol. 29, 224–227 (1999). 10583748 10.1046/j.1365-2672.1999.00602.x

[75] M. Wehrens, D. Ershov, R. Rozendaal, N. Walker, D. Schultz, R. Kishony, P. A. Levin, S. J. Tans, Size laws and division ring dynamics in filamentous *Escherichia coli* cells. Curr. Biol. 28, 972–979.e5 (2018). 29502951 10.1016/j.cub.2018.02.006

[76] L. Lacerda Mariano, M. A. Ingersoll, The immune response to infection in the bladder. Nat. Rev. Urol. 17, 439–458 (2020). 32661333 10.1038/s41585-020-0350-8

[77] K. Schaale, K. M. Peters, A. M. Murthy, A. K. Fritzsche, M. D. Phan, M. Totsika, A. A. B. Robertson, K. B. Nichols, M. A. Cooper, K. J. Stacey, G. C. Ulett, K. Schroder, M. A. Schembri, M. J. Sweet, Strain- and host species-specific inflammasome activation, IL-1β release, and cell death in macrophages infected with uropathogenic *Escherichia coli*. Mucosal Immunol. 9, 124–136 (2016). 25993444 10.1038/mi.2015.44

[78] C. Hamilton, L. Tan, T. Miethke, P. K. Anand, Immunity to uropathogens: The emerging roles of inflammasomes. Nat. Rev. Urol. 14, 284–295 (2017). 28266511 10.1038/nrurol.2017.25

[79] I. Demirel, A. Persson, A. Brauner, E. Särndahl, R. Kruse, K. Persson, Activation of the NLRP3 inflammasome pathway by uropathogenic *Escherichia coli* is virulence factor-dependent and influences colonization of bladder epithelial cells. Front. Cell. Infect. Microbiol. 8, 81 (2018). 29662840 10.3389/fcimb.2018.00081 PMC5890162

[80] S. Bouillot, S. Pont, B. Gallet, C. Moriscot, V. Deruelle, I. Attrée, P. Huber, Inflammasome activation by *Pseudomonas aeruginosa*’s ExlA pore-forming toxin is detrimental for the host. Cell. Microbiol. 22, e13251 (2020). 32779854 10.1111/cmi.13251

[81] G. C. Ulett, R. I. Webb, K. B. Ulett, X. Cui, W. H. Benjamin, M. Crowley, M. A. Schembri, Group B Streptococcus (GBS) urinary tract infection involves binding of GBS to bladder uroepithelium and potent but GBS-specific induction of interleukin 1α. J. Infect. Dis. 201, 866–870 (2010). 20132033 10.1086/650696

[82] M. Kaparakis-Liaskos, R. L. Ferrero, Immune modulation by bacterial outer membrane vesicles. Nat. Rev. Immunol. 15, 375–387 (2015). 25976515 10.1038/nri3837

[83] L. Chorro, Z. Li, L. Chu, S. Singh, J. Gu, J. H. Kim, K. Dutta, R. Pan, S. Kodali, D. Ndreu, A. Patel, J. C. Hawkins, C. Ponce, N. S. de Monerri, D. Keeney, A. Illenberger, C. H. Jones, L. Andrew, J. Lotvin, A. K. Prasad, I. Kanevsky, K. U. Jansen, A. S. Anderson, R. G. K. Donald, Preclinical immunogenicity and efficacy of optimized O25b O-antigen glycoconjugates to prevent MDR ST131 E. coli infections. Infect. Immun. 90, e0002222 (2022). 35311580 10.1128/iai.00022-22 PMC9022517

[84] K. A. Datsenko, B. L. Wanner, One-step inactivation of chromosomal genes in *Escherichia coli* K-12 using PCR products. Proc. Natl. Acad. Sci. U.S.A. 97, 6640–6645 (2000). 10829079 10.1073/pnas.120163297 PMC18686

[85] S. Sathiananthamoorthy, J. Malone-Lee, K. Gill, A. Tymon, T. K. Nguyen, S. Gurung, L. Collins, A. S. Kupelian, S. Swamy, R. Khasriya, D. A. Spratt, J. L. Rohn, Reassessment of routine midstream culture in diagnosis of urinary tract infection. J. Clin. Microbiol. 57, e01452-18 (2019). 30541935 10.1128/JCM.01452-18 PMC6425166

[86] I. Wiegand, K. Hilpert, R. E. W. Hancock, Agar and broth dilution methods to determine the minimal inhibitory concentration (MIC) of antimicrobial substances. Nat. Protoc. 3, 163–175 (2008). 18274517 10.1038/nprot.2007.521

[87] A. Ducret, E. M. Quardokus, Y. V. Brun, MicrobeJ, a tool for high throughput bacterial cell detection and quantitative analysis. Nat. Microbiol. 1, 16077 (2016). 27572972 10.1038/nmicrobiol.2016.77 PMC5010025

