# Supplementary material for: A human urothelial microtissue model reveals shared colonization and survival strategies between uropathogens and commensals
Source: Sci Adv. 2023 Nov 8;9(45):eadi9834. doi: 10.1126/sciadv.adi9834 (PMC10631729; doi:10.1126/sciadv.adi9834)
Supplement: Supplementary file 1 — Figs. S1 to S7 Table S1 [file sciadv.adi9834_sm.pdf]

Science Advances
AAAS

Supplementary Materials for

A human urothelial microtissue model reveals shared colonization and survival strategies between uropathogens and commensals

Carlos Flores *et al.*

Corresponding author: Jennifer L. Rohn, j.rohn@ucl.ac.uk

Sci. Adv. **9**, eadi9834 (2023)
DOI: 10.1126/sciadv.adi9834

This PDF file includes:

Figs. S1 to S7
Table S1

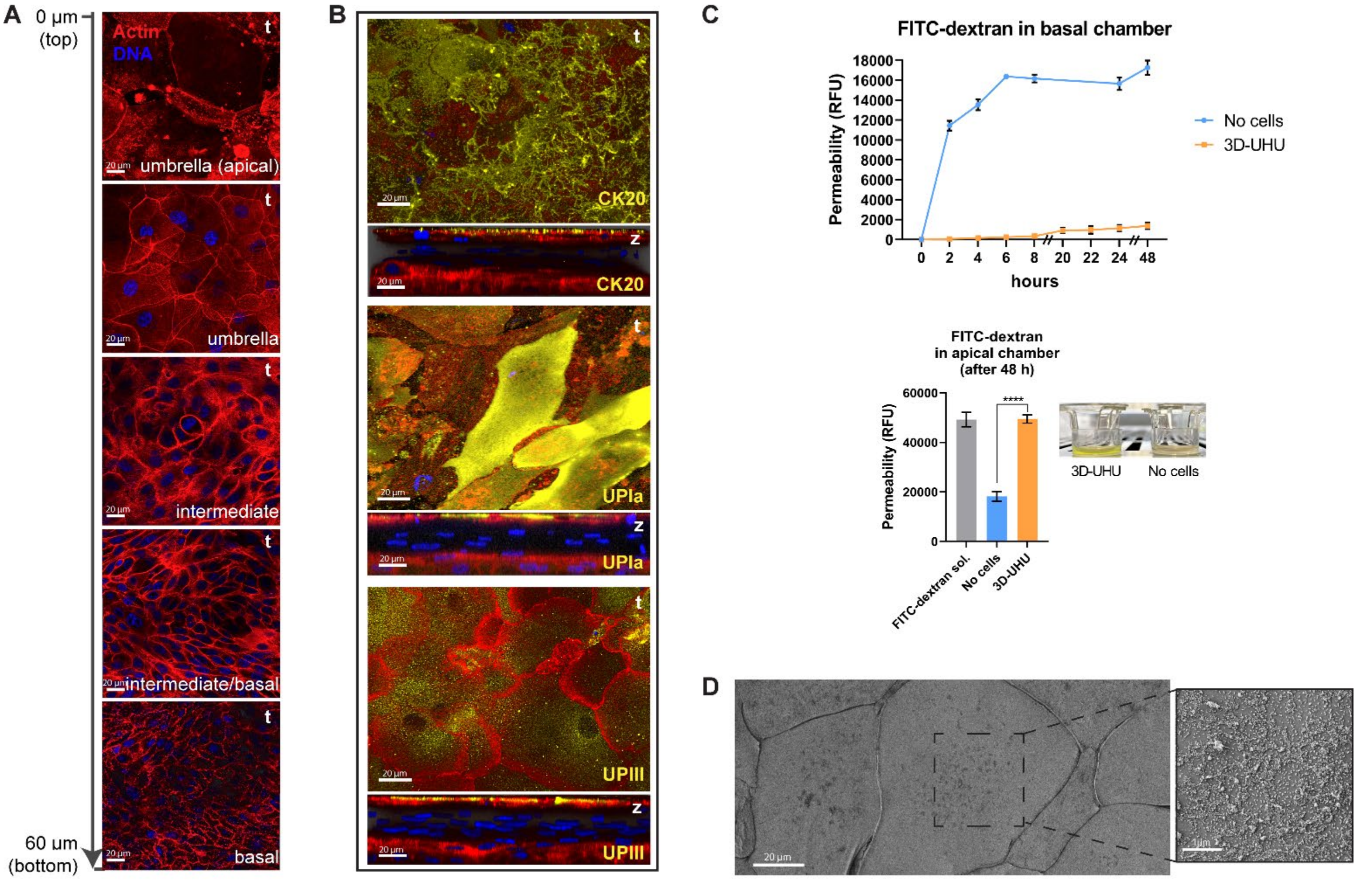

Fig. S1. 3D-UHU stratification and differentiation.

(**A**) 3D-UHU top-down (t) cross sections from top (0 μm) to bottom (60 μm) depicting the different cell morphologies of the main urothelial sublayers (umbrella, intermediate, basal). Blue, DNA; Red, F-actin. (**B**) Expression of terminal differentiation markers (yellow) cytokeratin 20 (CK20) and uroplakins Ia (UPIa) and III (UPIII) by apical umbrella cells. (**C**) FITC-dextran (4 kDa) translocation assay using Transwell™ inserts without cells and with 3D-UHU. Fluorescence was measured in the basal (top panel) and apical (bottom panel) chambers over 48 h (****$P < 0.0001$); the latter was compared with FITC-dextran stock solution (1 mg/ml). Image inset to the right shows FITC-dextran retention by 3D-UHU insert after 48 h (in bright yellow). (**D**) 3D-UHU topography showing tight junctions, GAG polysaccharides and microplicae/hinges on umbrella cells. Confocal (A, B) and SEM (D) images representative of a minimum of 4 independent biological replicates per strain ($N \geq 4$).

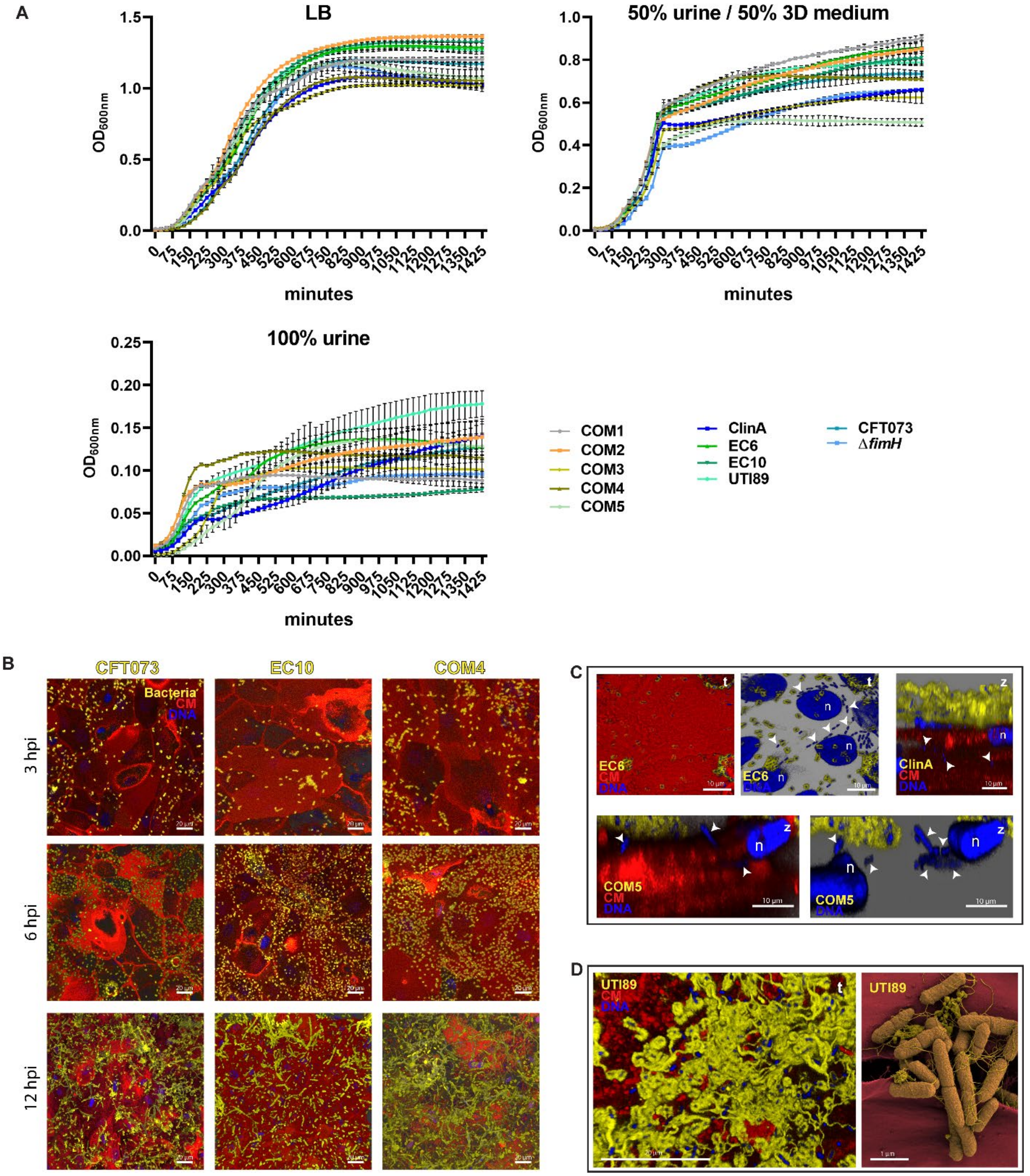

Fig S2. Growth fitness and 3D-UHU colonization by UPEC and commensals.

(**A**) Growth curves of *E. coli* strains at 37°C in LB media, 50% urine/50% CnT-Prime-3D mixed medium, or 100% human urine (initial inoculum OD_{600nm} 0.006 ± 0.003). Results are expressed as mean ± SD of biological triplicates. (**B**) Top-down views of bacterial growth at the apical surface of 3D-UHU over time, at 3, 6 and 12 hpi. Yellow, extracellular bacteria; Red, cell

membrane (CM); Blue, DNA of host nuclei and bacteria. (**C**) Isolated intracellular bacteria (arrowheads) by UPEC clinical isolates EC6 and ClinA, and commensal COM5. Staining as B. n, depicts host cell nuclei. t, top-down view; z, side view. (**D**) Biofilm-like aggregates of UTI89. Confocal (B, C, D, left) and SEM (D, right) images representative of a minimum of 4 independent biological replicates per strain ($N \geq 4$).

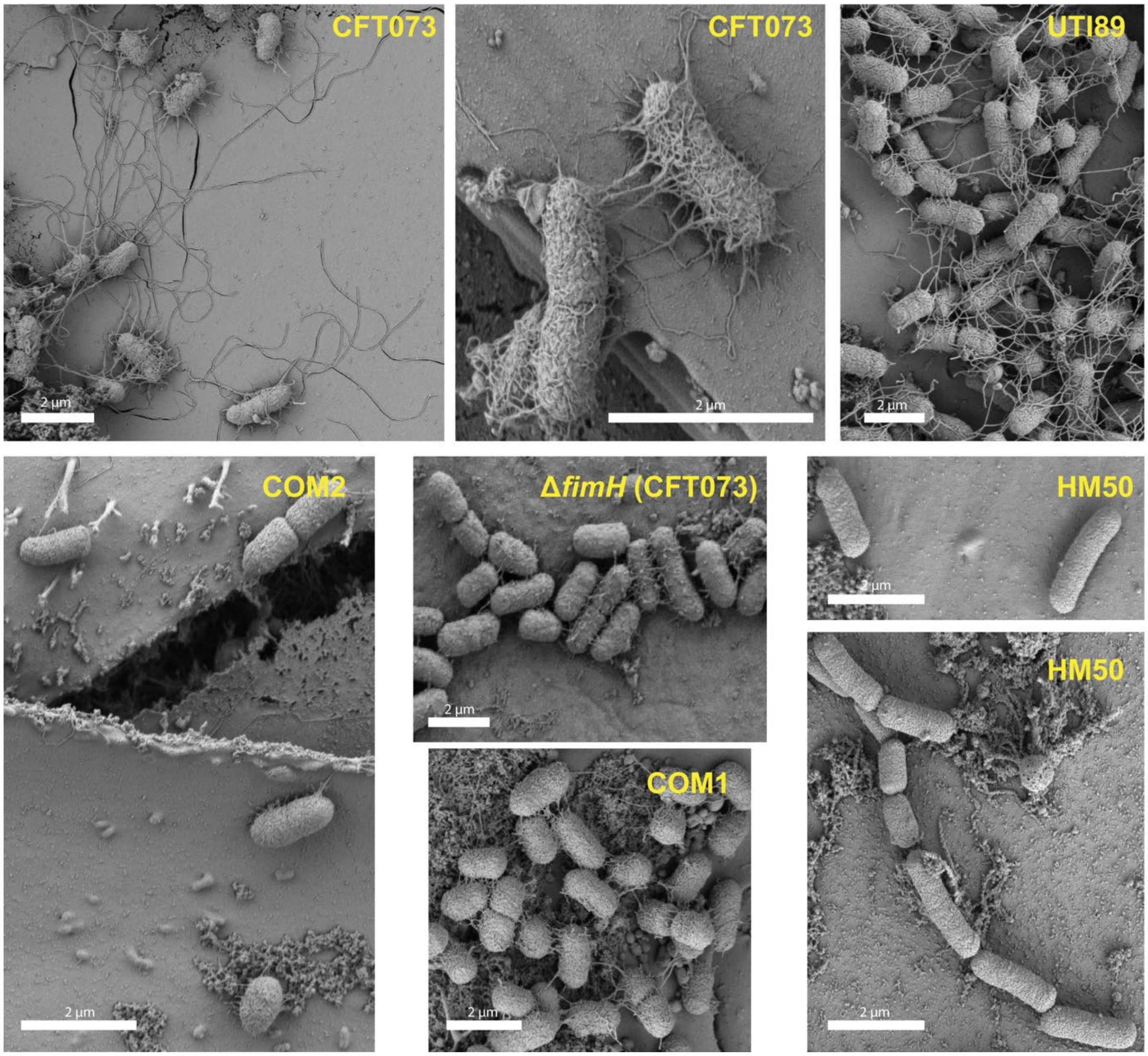

Fig. S3. UPEC and non-uropathogenic *E. coli* surface and piliation after infection.

SEM micrographs depicting bacterial surface and piliation of UPEC and nonpathogenic *E. coli* 12 hpi in the 3D-UHU model. Images are representative of a minimum of 4 independent biological replicates per strain ($N \geq 4$).

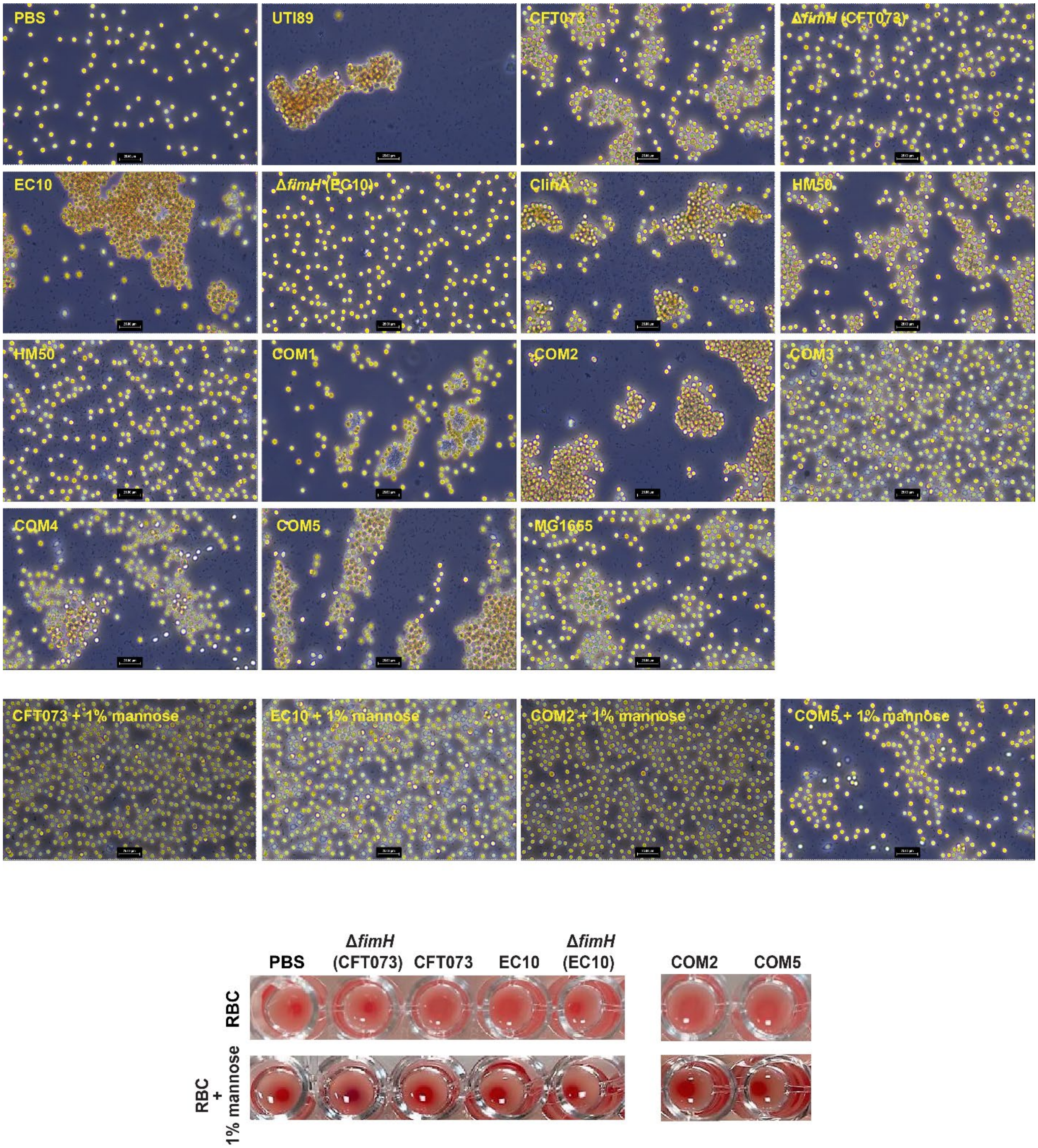

Fig. S4. Hemagglutination assays with commensal and uropathogenic *E. coli*.
Brightfield images of hemagglutination of 5% guinea pig red blood cells (RBC) by commensal and uropathogenic *E. coli* strains after 30 min incubation at room temperature, with and without 1% mannose. Bottom panel compares hemagglutination between Δ*fimH* mutants and respective parental strains, as well as the commensals COM2 and COM5, in microtiter plates. Scale bars, 25 μm.

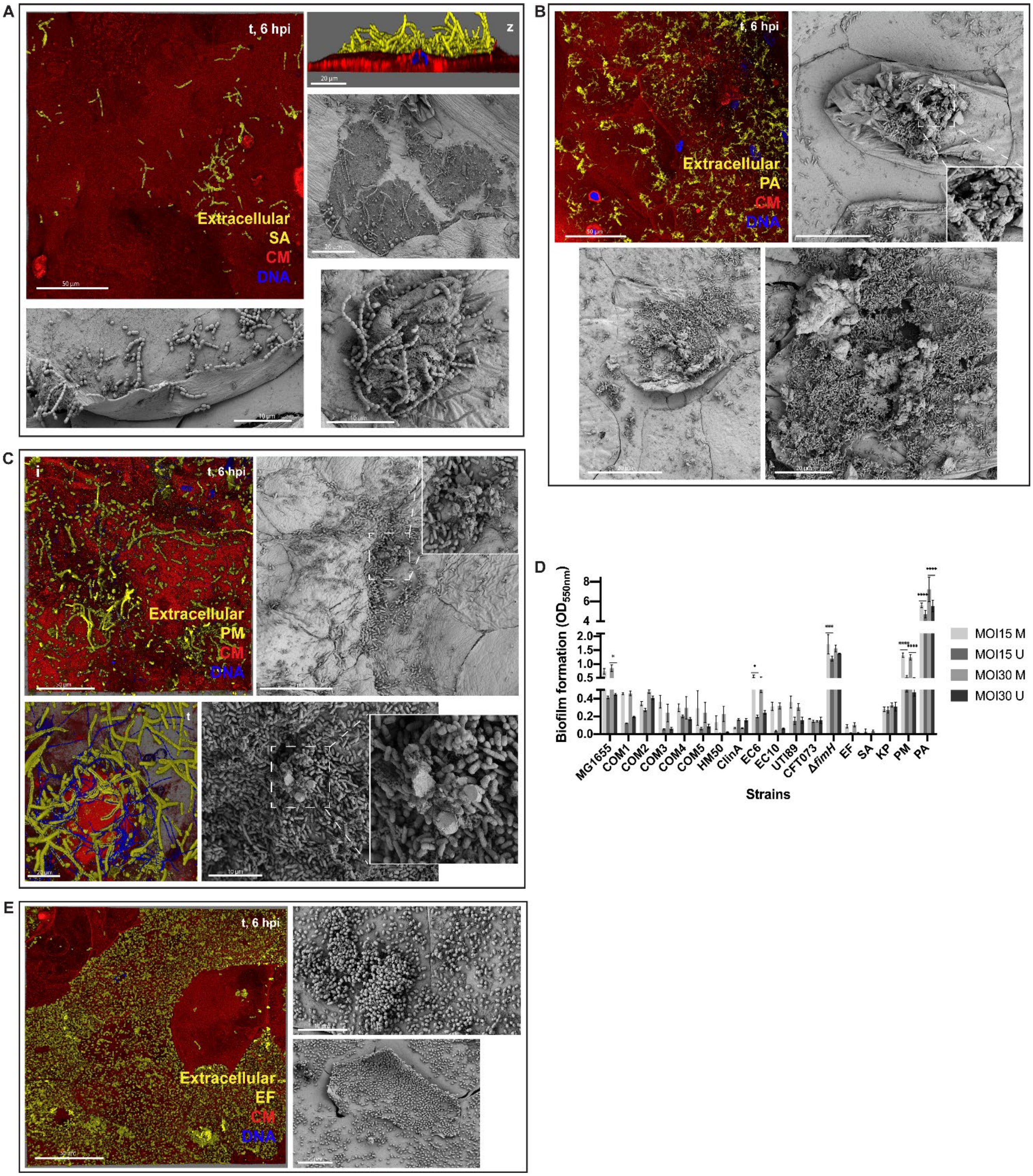

Fig. S5. Morphology and colonization strategies by non-UPEC uropathogens in the urothelial microenvironment.

(**A**) *S. agalactiae* (SA) in discrete regions of the urothelial surface, associated with damaged upper cell host membranes (right). Cocci and chains underneath cell being exfoliated or dying

(bottom left and right, respectively). (**B**) *P. aeruginosa* (PA) small biofilm aggregates 6 hpi, and larger at 12 hpi with incorporation of exfoliating cell debris and crystalline structures (right and bottom). (**C**) *P. mirabilis* (PM) chains and filamentous forms, and interjunctional crystalline biofilm aggregates in the inflamed microtissue. (**D**) Assessment of biofilm formation by crystal violet assay using Calgary system after 24 h of bacterial incubation in 25% urine (U) and LB media (M), at MOI 15 and 30. OD at 550 nm is presented as mean ± SE of 2 biological and 4 technical replicates (*$P < 0.1$; **$P < 0.01$; ***$P < 0.001$; ****$P < 0.0001$). (**E**) *E. faecalis* (EF) spread on the urothelial surface 6 hpi, heavily colonized umbrella cells being exfoliated (right bottom) and biofilm-like aggregates (right top). Staining as in fig. S2. t, top-down view; z, side view. Confocal (A top, B-E left) and SEM (A-E right and bottom) images are representative of a minimum of 4 independent biological replicates per strain ($N \geq 4$).

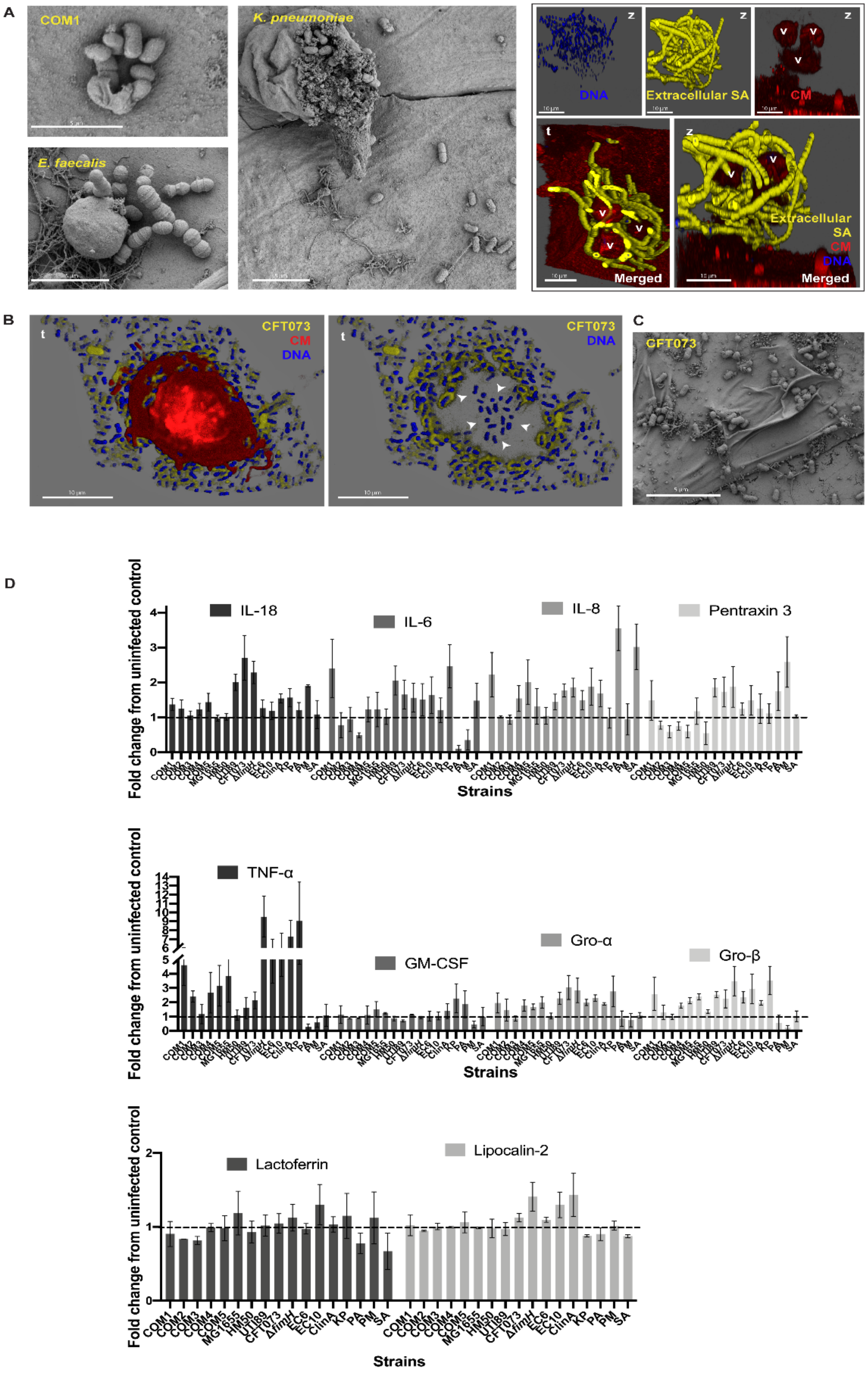

Fig. S6. 3D-UHU tissue responses to infection.

(**A**) Uropathogens (*E. faecalis*, *K. pneumoniae*) and commensal *E. coli* (COM1) erupting from vesicle-like structures/host cell fragments, and *S. agalactiae* (SA) chains surrounding vesicle-like

structures. Staining as in fig. S2. t, top-down view; z, side view. (**B**) Top-down view of a cytospun shed cell fragment recovered from the supernatant of an infected 3D-UHU, containing intracellular UPEC CFT073 (arrowheads). (**C**) Umbrella cell membrane ruffling 12 hpi with CFT073. (**D**) Fold changes, compared to uninfected controls, of cyto/chemokine production by 3D-UHU after 12 hpi with non-pathogenic bacteria and uropathogens. Dashed lines represent the mean for uninfected controls, considering three biological and technical replicates. Confocal (A right and B left) and SEM (A left and C) images are representative of a minimum of 4 independent biological replicates per strain ($N \geq 4$).

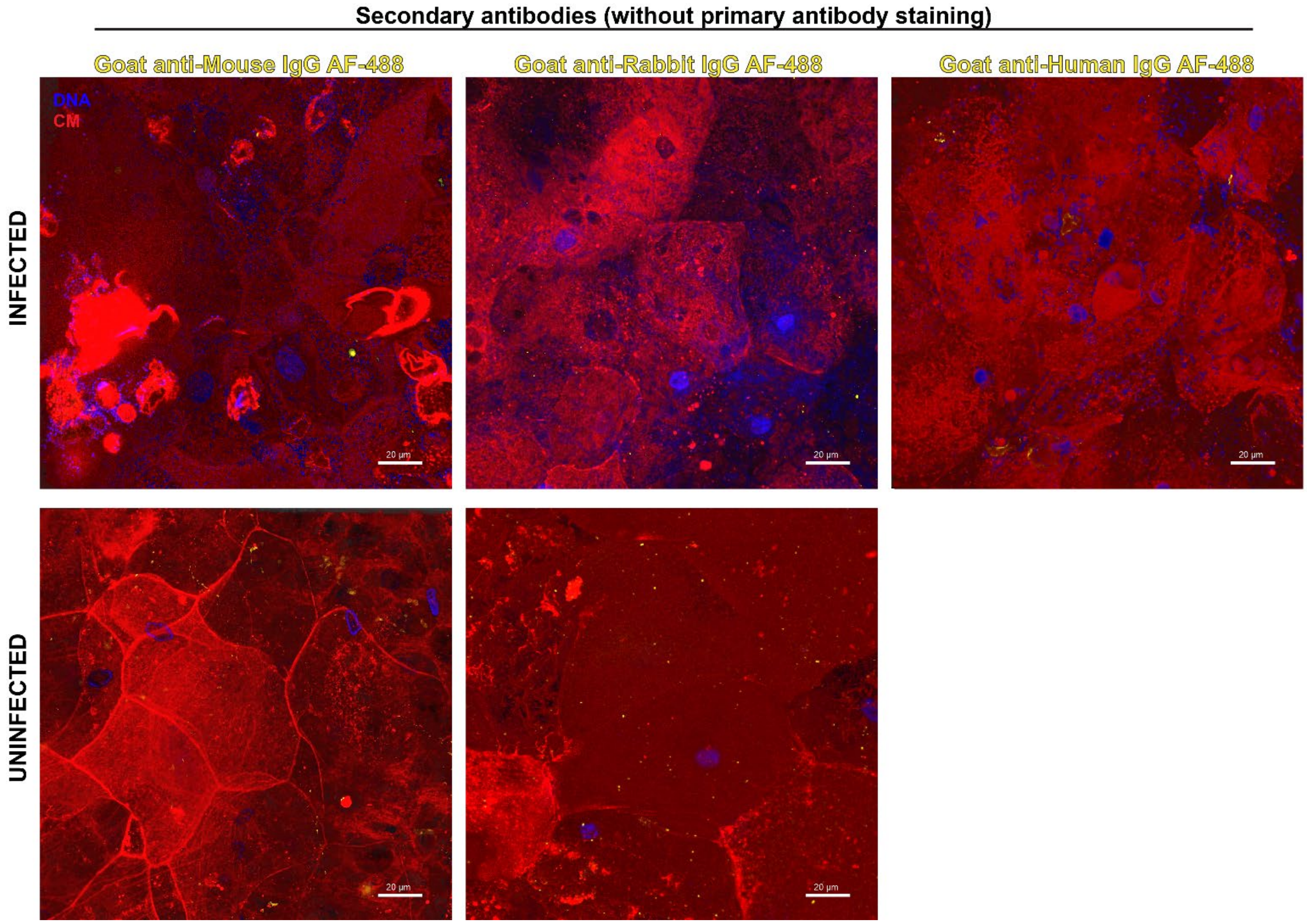

Fig. S7. Controls of 3D-UHU immunostaining without primary antibody.

Top-down views of infected (CFT073, right; *P. mirabilis*, middle; EC10, left) and uninfected 3D-UHU model after immunostaining without using primary antibodies, but with the use of secondary antibodies alone at 1:300 dilutions (more details in *Material and Methods*). Red, cell membrane (CM); Blue, DNA of host nuclei and bacteria.

STRAIN	ORIGIN	MLST	O-ANTIGEN	FIMH
**CFT073**	Pyelonephritis patient	73	O6	10
**UTI89**	Cystitis patient	95	O18	18
**EC10**	Recurrent UTI patient	131	O25b	30
**EC6**	Recurrent UTI patient	131	O25b	30
**CLINA**	Recurrent UTI patient	101	O96	34
**COM1**	Healthy volunteer	394	O17/O77	30
**COM2**	Healthy volunteer	155	O58	30
**COM3**	Healthy volunteer	46	O9	34
**COM4**	Healthy volunteer	140	O50/O2	15
**COM5**	Healthy volunteer	69	O15	27
**HM50**	Asymptomatic bacteriuria	73	Nt	12
**MG1655**	Lab strain	10	OR	27

Table S1. Genotyping of the *E. coli* strains used in this study.

Information about Multilocus Sequence Typing (MLST), O-Antigen and FimH variants retrieved from the *E. coli* strains tested.
